# Was bewegt Lehrpersonen während der Schulschließungen? – Eine Analyse der Kommunikation im Twitter-Lehrerzimmer über Chancen und Herausforderungen digitalen Unterrichts

**DOI:** 10.1007/s11618-021-01013-8

**Published:** 2021-04-01

**Authors:** Tim Fütterer, Emely Hoch, Kathleen Stürmer, Andreas Lachner, Christian Fischer, Katharina Scheiter

**Affiliations:** 1 Hector-Institut für Empirische Bildungsforschung, Universität Tübingen, Europastraße 6, 72072 Tübingen, Deutschland; 2 Multiple Repräsentationen, Leibniz-Institut für Wissensmedien, Tübingen, Deutschland; 3 Institut für Erziehungswissenschaft, Universität Tübingen, Tübingen, Deutschland; 4 Fachbereich Psychologie, Universität Tübingen, Tübingen, Deutschland

**Keywords:** Corona-Pandemie, Twitter, Lehrpersonen, Digitaler Fernunterricht, Chancen und Herausforderungen, Corona-Pandemic, Twitter, Teacher, Digital Distance Learning, Chances and Challenges

## Abstract

Während der durch die Corona-Pandemie bedingten Schulschließungen im März 2020 führten viele Schulen Fernunterricht ein, der häufig ohne wirkliche Vorbereitung als digitaler Unterricht organisiert wurde. Daraufhin war ein verstärkter Austausch unter Lehrpersonen in Online-Communities zu erwarten. Eine Analyse der Kommunikation der Online-Community Twitter-Lehrerzimmer erlaubte Einblick in aktuelle Themen und ermöglichte zudem den Vergleich von Themen vor und während der Schulschließungen. Zur Identifikation von Themen wurden computerlinguistische Analysemethoden basierend auf 128.422 Tweets sowie eine qualitative Inhaltsanalyse von 270 Tweets durchgeführt. Es zeigte sich, dass Themen wie (a)synchroner digitaler Unterricht bereits vorher besprochen, während der Schulschließungen jedoch häufiger und breiter thematisiert wurden. Das Twitter-Lehrerzimmer wurde für gegenseitige Unterstützung sowie den Austausch über drängende Herausforderungen genutzt wie etwa die Verfügbarkeit geeigneter (datenschutzkonformer) Software. Die Ergebnisse legen somit Defizite des Digitalisierungsprozesses aus der Perspektive Twitter-affiner Lehrpersonen in Deutschland offen und zeigen das Potenzial von Online-Communities für Austausch und Vernetzung.

## Abstract (en)

Many schools introduced distance learning as a result of the school closings due to the Corona pandemic in March 2020. Such instruction was often organized digitally without much prior preparation. As a result, an increased exchange between teachers in online communities was to be expected. Analyzing the communication of the online community Twitter-Lehrerzimmer provided insight into topics and allowed to compare topics that were discussed before and during school closings. To identify topics, we applied computational linguistic analysis methods on 128,422 tweets and qualitative content analysis of 270 tweets. The results indicated that topics such as (a)synchronous digital teaching had already been discussed previously but was addressed more often and in more breadth during school closings. The Twitter-Lehrerzimmer was used for mutual support and exchange on urgent challenges such as the availability of high-quality software (compliant with data protection). The results reveal deficits in the German digitalization process from the perspective of teachers using Twitter and show the potential of online communities for information exchange and networking.

## Einleitung

Die durch die Corona-Pandemie bedingten Schulschließungen im März 2020 haben eindrücklich vor Augen geführt, wie alternativlos das schulische Bildungssystem in Deutschland zu traditionellem Unterricht ist. Innerhalb weniger Tage mussten Schulen Ersatz für den traditionellen Präsenzunterricht finden und umsetzen, um der Forderung der Kultusministerkonferenz (KMK) gerecht zu werden, den Unterricht weitestgehend aufrechtzuerhalten und das bestmögliche Bildungsangebot zu bieten (KMK 2020b). Dabei erschien es vielen Schulen naheliegend, digitalen Fernunterricht einzuführen (Vodafone Stiftung Deutschland 2020). Allerdings sahen sich Lehrpersonen, Schülerinnen und Schüler sowie Erziehungsberechtigte vor große Herausforderungen gestellt: Die wenigsten Schulen verfügten über die notwendige digitale Infrastruktur um kohärente Bildungsangebote bereitzustellen (z. B. Cloudlösungen, die ein Teilen von Lern- und Arbeitsmaterialien ermöglichen), Lehrpersonen waren oftmals nur unzureichend auf den Einsatz digitaler Medien – im Unterricht und noch weniger im Kontext des Fernunterrichts – vorbereitet, Schülerinnen und Schüler verfügten nicht über die notwendigen (Medien‑)Kompetenzen sich selbstreguliert Inhalte mithilfe digitaler Lernmedien anzueignen und Erziehungsberechtigte verzweifelten vor der Aufgabe, die nötige Infrastruktur zuhause bereitzustellen und ihre Kinder im Lernprozess anzuleiten und zu unterstützen. Diese Darstellung mag übermäßig dramatisierend wirken, beschreibt aber vielerorts die Situation sehr treffend, wie sie sich auch in der „JIMplus Corona Studie“ des medienpädagogischen Forschungsverbund Südwest (2020) widerspiegelt.

Eine wesentliche Ursache für diese Situation liegt in dem jahrelangen Versäumnis, das deutsche Bildungssystem auf das Thema Digitalisierung im Kontext schulischen Lernens angemessen vorzubereiten (Scheiter und Lachner 2019). Neben mangelnder Infrastruktur verfügen die wenigsten Schulen über strukturelle und didaktische Konzepte zum Unterrichten mit digitalen Medien. Weiter gibt es kaum curricular motivierte oder empirisch geprüfte digitale Unterrichtsmaterialien, die zu einem kohärenten digitalen Bildungsangebot beitragen könnten (Scheiter und Lachner 2019). Darüber hinaus stellt das Thema Unterrichten mit digitalen Medien bisher – wenn überhaupt – einen eher sporadischen Inhalt für die Professionalisierung von Lehrpersonen in der Lehrerbildung dar. Die durch die Corona-Pandemie notwendigen Schulschließungen können demnach als Belastungstest der Machbarkeit und Funktionsfähigkeit des deutschen Schulsystems bezüglich digitalen Unterrichtens betrachtet werden. Doch wie wird das von den eigentlichen Akteuren, die digitalen Fernunterricht bereitstellen müssen und mussten – den Lehrpersonen – wahrgenommen? Bieten die Schulschließungen neben Herausforderungen vielleicht auch Chancen für das digitale Unterrichten?

Erste empirische Befunde legen nahe, dass digital gestützter Unterricht in Deutschland bisher vor allem denjenigen „Medienenthusiasten“ überlassen war, die sich aus persönlicher Überzeugung im Selbststudium in die Thematik eingearbeitet haben (Backfisch et al. 2021). Solche medienaffinen Lehrpersonen nutzen digitale Medien nicht nur für den Unterricht, sondern auch für ihre eigene Weiterbildung und den informellen Austausch mit Gleichgesinnten, wie beispielsweise über die Online-Plattform Twitter (Fischer et al. 2019; Visser et al. 2014). Gerade während der Schulschließungen, also als der persönliche Austausch im realen Lehrerzimmer wegfiel, liegt es nahe, zu vermuten, dass medienaffine Lehrpersonen, aber auch weniger erfahrene Lehrpersonen auf solche Plattformen zurückgreifen, um sich über die Anforderungen des digitalen Fernunterrichts auszutauschen.

Aus Forschungssicht bietet daher die Analyse von Twitterdaten das Potenzial zu untersuchen, welche Themen Lehrpersonen vor und während der Schulschließungen bewegten, ohne dass großangelegte Fragebogeninstrumente implementiert werden müssten. Unter der Nutzung computerlinguistischer Methoden, wurde in dieser Studie anhand von Twitterbeiträgen untersucht, (1) welche Themen die Lehrpersonen im Zuge der Schulschließungen insbesondere mit Fokus auf das Unterrichten mit digitalen Medien beschäftigten sowie (2) welche Chancen und Herausforderungen Lehrpersonen mit der neuen Situation des digitalen Fernunterrichts verbanden.

### Einsatz digitaler Medien an Schulen in Deutschland und die Rolle der Lehrpersonen vor der Corona-Pandemie

Bereits vor der Corona-Pandemie fand in Deutschland die Nutzung digitaler Medien im Unterricht kaum statt. Im Rahmen der internationalen Vergleichsstudie International Computer and Information Literacy Study (ICILS) wurden Lehrpersonen aus Klassen der achten Jahrgangsstufe hinsichtlich der Häufigkeit der Nutzung von Computern im Unterricht befragt. Deutschland belegte hinsichtlich der Häufigkeit des Computereisatzes im Vergleich von 21 Bildungssystemen den letzten Platz (Bos et al. 2014). Eine erneute Befragung 2018 zeigt hier kaum eine Verbesserung im Ranking (Eickelmann et al. 2019). Die mangelnden (infra)strukturellen Voraussetzungen an Schulen sind eine notwendige, aber nicht hinreichende Erklärung für diese eingeschränkte Nutzung (Scheiter 2016; Scheiter und Lachner 2019). Gerade Lehrpersonen kommt eine entscheidende Rolle beim Unterrichten mit digitalen Medien zu (Drossel und Eickelmann 2018; Eickelmann und Drossel 2020). Nach dem aktuellem Verständnis über die Voraussetzungen zur lernwirksamen Gestaltung von Unterricht kann davon ausgegangen werden, dass Lehrpersonen professionelle Kompetenzen, d. h. Professionswissen, motivationale Voraussetzungen, Einstellungen und selbstregulatorischen Fähigkeiten, aufweisen müssen, um digitale Medien didaktisch sinnvoll für das Unterrichten zu nutzen (Baumert und Kunter 2006; Stürmer und Lachner 2017). Laut ICILS 2018 schätzten 78,9 % der deutschen Lehrpersonen ein, dass sie digitalen Unterricht planen können (ICILS 2013: 67,0 %). Dieser Anteil ist zwar gestiegen, liegt aber immer noch unter dem internationalen Mittelwert (83,9 %). Belastbare Erkenntnisse zu den faktischen (nicht-selbsteingeschätzten) Kompetenzen zum lernwirksamen Unterrichten mit digitalen Medien liegen darüber hinaus erst vereinzelt vor (z. B. zum technologisch-pädagogischen Wissen: Lachner et al. 2019).

Eher verweisen erste Befunde darauf, dass Lehrpersonen in Deutschland ungünstige Voraussetzungen für das Unterrichten mit digitalen Medien mitbringen. So zeigt sich beispielsweise, dass sie – wenn sie digitale Medien im Unterricht nutzen – bisherige Unterrichtsverfahren oder analoge Medien lediglich ersetzen (Backfisch et al. 2020). Die Ergebnisse aus ICILS zeigen darüber hinaus, dass Lehrpersonen in Deutschland dem Einsatz digitaler Medien im Unterricht ein deutlich geringeres Lernpotenzial zuschreiben als dies in anderen Ländern der Fall ist, auch wenn die Werte in 2018 gegenüber 2013 höher ausfielen. Dies ist problematisch, da solche Nützlichkeitsüberzeugungen hinsichtlich digitaler Medien einen wesentlichen Prädiktor für die Häufigkeit der Mediennutzung und vor allem auch für die Qualität des resultierenden Unterrichts darstellen (Backfisch et al. 2020). Lehrpersonen in Deutschland haben dagegen häufiger als Lehrpersonen anderer Länder Bedenken gegenüber dem Medieneinsatz. Laut ICILS 2013 (die Variablen wurden 2018 nicht erhoben) werden in fast keinem Teilnehmerland Aussagen zu organisatorischen Problemen und Problemen beim Umgang mit Informationsquellen häufiger bejaht als in Deutschland.

Insgesamt verweisen die bisherigen Befunde auf eine sehr ungünstige Ausgangslage für die durch die Corona-Pandemie über Nacht notwendig gewordene Digitalisierung im schulischen Bildungsbereich.

### Die Corona-Pandemie und damit verbundene Schulschließungen

**Figure 1:**
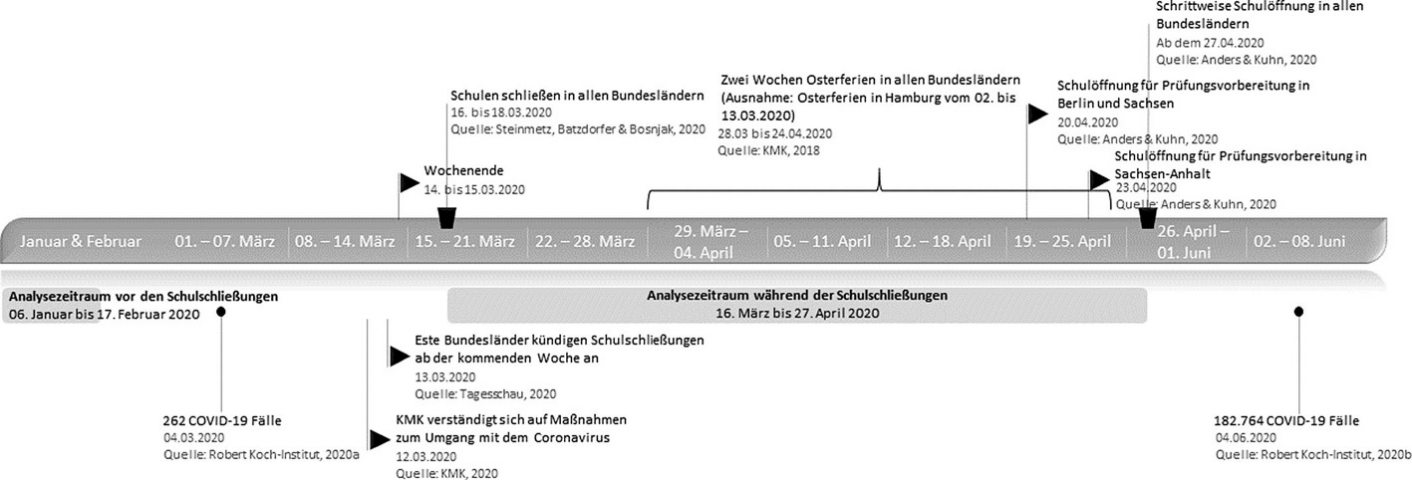

Zu Beginn des Jahres 2020 breitete sich das Coronavirus (SARS-CoV-2) global aus und entwickelte sich zu einer Pandemie. Da zu diesem Zeitpunkt weder ein Impfstoff noch spezifische Medikamente zur Behandlung einer COVID-19 Erkrankung verfügbar waren, wurden bundes- sowie weltweit Maßnahmen zum Infektionsschutz ergriffen, die darauf abzielten, soziale Kontakte drastisch zu beschränken. Daher ordneten die Landesregierungen in Einvernehmen mit dem Bund im März 2020 kurzfristig eine bundesweit angelegte Schließung aller Schulen an (siehe Abb. 1 für den Schließungsverlauf). Aufgrund der Kurzfristigkeit der Schulschließung gab es annähernd keine Vorbereitungszeit für die Umstellung von Präsenz- auf Fernunterricht. Neben Unterrichtsausfällen reichten Unterrichtsangebote von dem Verschicken von Briefen oder E‑Mails mit Arbeitsaufträgen bis hin zu Videokonferenzen und digitalisierten Lehrangeboten in virtuellen Klassenzimmern. Eine aktuelle Umfrage zeigt, dass Lehrpersonen von allgemeinbildenden Schulen, die bereits vor den Schulschließungen verstärkt digitale Medien eingesetzt hatten, angaben, besser auf die Situation vorbereitet gewesen zu sein (Vodafone Stiftung Deutschland 2020).

Die Schulschließungen zwangen Lehrpersonen allerdings nicht nur routinierte Verhaltensweisen des Unterrichtens aufzugeben. Auch der informelle Austausch und Absprachen im Kollegium waren deutlich erschwert. Nach den Erkenntnissen der Vodafone Stiftung (2020) koordinierten sich viele Lehrpersonen eigenständig untereinander. Anzunehmen ist, dass Lehrpersonen darüber hinaus verstärkt Online-Plattformen als Möglichkeit des Austausches mit Kolleginnen und Kollegen anderer Schulen und als Möglichkeit für informelles Lernen nutzten. Damit erscheinen Plattformen, wie Twitter, geeignet, um Einblick in relevante Themen zu bekommen, die Lehrpersonen in Zeiten der Schulschließungen bewegten.

### Twitter und die Online-Community Twitter-Lehrerzimmer

Twitter (https://twitter.com) ist ein Mikrobloggingdienst, bei welchem registrierte Nutzerinnen und Nutzer (*User*) kurze (d. h. 280 Unicode-Zeichen umfassende) Beiträge (*Tweets*) veröffentlichen. Tweets können mit spezifischen Themenfeldern (durch Hashtags [#]) oder bestimmten Usern (durch Verlinkungen [@]) verknüpft werden. Dabei können User die Tweets anderer User abonnieren (*follow*; jemandem folgen). Für Tweets können *Likes* vergeben (als Ausdruck der Zustimmung oder des Gefallens) und/oder *Comments* hinzugefügt werden (um den Tweet zu kommentieren). Zudem können Tweets als *Retweets* veröffentlicht werden (um einen Ursprungstweet kommentiert oder unkommentiert mit den eigenen Followern zu teilen). Die Analyse von Twitter-Daten wird in den Sozialwissenschaften zunehmend in der Forschung verwendet, um aktuelle Debatten und deren Entwicklung über die Zeit, deren Rezeption in den Medien sowie Einstellungen von Personen und Gruppen zu bestimmten Themen zu untersuchen (McCormick et al. 2017). Auch Lehrpersonen nutzen soziale Netzwerke wie Twitter seit vielen Jahren zunehmend als Plattform für informellen Austausch (Rosenberg et al. 2020; Trust 2012; Trust et al. 2016). Dabei spielen insbesondere der niedrigschwellige Zugang zu Informationen und der interaktive Charakter der Plattform eine Rolle (Carpenter und Krutka 2015). Im Vergleich zu traditionellen Lehrerzimmern konnte Twitter insbesondere auch während der Schulschließungen als Plattform genutzt werden (Trust et al. 2020). Hierfür werden in Deutschland überwiegend die beiden zentralen Hashtags #twitterlehrerzimmer und #twlz genutzt. Mitglieder, welche innerhalb dieser Hashtags Twitterbeiträge verfassen, teilen, kommentieren, liken oder lesen, können als Online-Community beziehungsweise als eine Art virtuelles Lehrerzimmer aufgefasst werden, in dem sich User und insbesondere Lehrpersonen informell zu schulbezogenen Themen aller Art austauschen und informieren (Gewerkschaft für Erziehung und Wissenschaft 2019). Da sich diese Community insbesondere auch mit Veränderungen, Chancen und Herausforderungen von Digitalisierungsprozessen beschäftigt, erscheint es lohnend, diese Twitter-Daten zu nutzen und bezüglich diskutierter Themen während der Schulschließungen auszuwerten. Die durch die Hashtags #twitterlehrerzimmer und #twlz abgebildete Community wird im Folgenden zur Vereinfachung nur noch als Online-Community Twitter-Lehrerzimmer bezeichnet.

### Überblick über die Studie und Fragestellungen

Vor dem Hintergrund der Schulschließungen, welche als Konsequenz eine radikale Disruption von Unterrichtshandeln bedeuteten, untersuchten wir anhand von Twitterdaten, wie und über welche Themen sich Lehrpersonen austauschten und wie sich diese Themen aufgrund der Schulschließungen in Zeiten der Corona-Pandemie veränderten.

#### F1

Welche Themen waren in der Community Twitter-Lehrerzimmer während der bundesweiten Schulschließungen drängend (z. B. bezüglich digitalen Unterrichts)?

Es sollte betrachtet werden, über welche Themen sich Lehrpersonen in der Twitter-Community austauschten, welches dabei die besonders drängenden Themen waren, also welche Themen besonders häufig auftraten und wie diese Themen zueinander in Beziehung standen. Dabei gingen wir davon aus, dass sich Themen bezüglich digitalen Unterrichts besonders salient zeigten.

#### F2

Wie unterschieden sich die Themen im Twitter-Lehrerzimmer vor und während der bundesweiten Schulschließungen (z. B. bezüglich digitalen Unterrichts)?

Diese Forschungsfrage nahm den Vergleich von Themen in den Fokus, die vor und während der Schulschließungen während der Corona-Pandemie von der Twitter-Community diskutiert wurden. Auch hier nahmen wir an, dass Themen bezüglich digitalen Unterrichts verstärkt während der Schulschließungen diskutiert worden sind.

#### F3

Welche Chancen und Herausforderungen wurden im Twitter-Lehrerzimmer (z. B. bezüglich digitalen Unterrichts) während der bundesweiten Schulschließungen thematisiert?

Während der Schulschließungen erschien es vielen Schulen naheliegend, digitalen Fernunterricht einzuführen. Es sollte analysiert werden, welche Chancen und Herausforderungen in diesem Zusammenhang in der Online-Community Twitter-Lehrerzimmer diskutiert wurden.

## Methode

### Daten

Es wurden alle verfügbaren Tweets, die auf Twitter unter den Hashtags *#twitterlehrerzimmer* und/oder *#twlz* im Zeitraum zwischen dem 01. November 2019 (00:00 Uhr) und dem 03. Juni 2020 (12:00 Uhr) veröffentlicht worden waren, mit dem R‑Paket *rtweet *(Version 0.7.0; Kearney 2019) am 03. Juni 2020 zwischen 16:30 und 18:30 Uhr heruntergeladen. Der Rohdatensatz bestand aus 183.686 Tweets. Die beiden Hashtags #twitterlehrerzimmer und #twlz wurden ausgewählt, da diese häufig von Lehrpersonen im deutschsprachigen Raum genutzt werden, um unterrichts- und schulbezogene Themen zu diskutieren. Zudem wird diese Twitter-Community durch ein automatisiertes Computerprogramm (Bot *Tw!tterlehrerzimmer*) abgebildet. Ein Bot ist ein Computerprogramm, dessen einzige Funktion darin besteht, Tweets zu retweeten, die mindestens ein Hashtag aus einer Gruppe bestimmter Hashtags enthalten. Der Bot *Tw!tterlehrerzimmer* retweetet beispielsweise alle Tweets, die einen der Hashtags #twitterlehrerzimmer, #twlz oder #twitterlz enthalten. Es ist nicht möglich auf Twitter einzelnen Hashtags direkt zu folgen. User können jedoch diesem Bot folgen, um alle Tweets mit den obigen Hashtags angezeigt zu bekommen und zu lesen. Das Twitter-Lehrerzimmer definiert sich somit aus der Gruppe der Twitter-User, die mit einem dieser Hashtags twittern oder diesem Bot folgen. In explorativen Analysen zur Häufigkeit der Verwendung dieser drei Hashtags zeigte sich, dass das Hashtag #twlz am häufigsten und bei Verwendung weiterer Hashtags in einem Tweet am häufigsten mit dem Hashtag #twitterlehrerzimmer verwendet wurde. Kaum und insbesondere nicht ohne einen der anderen Hashtags wurde das Hashtag #twitterlz verwendet. Somit fiel die Auswahl der Hashtags auf #twitterlehrerzimmer und #twlz. Weitere prominente Hashtags im Bildungsbereich, die lokale Communities oder Communities mit spezifischen Zielen adressierten wurden bewusst nicht berücksichtigt (z. B. #edupnx, #eduBW). Das Twitter-Lehrerzimmer kann damit als Pendant zu einem analogen Lehrerzimmer gesehen werden, welches Raum zu einem geographisch überregionalen Austausch über das eigene Kollegium hinaus ermöglicht, ohne dass dabei Themen vorab programmatisch gesetzt oder lokale Themen in den Fokus gerückt werden.

#### Datenaufbereitung

Eine detaillierte Beschreibung der Datenaufbereitung befindet sich in Anhang A1. Zunächst wurden diejenigen doppelten Tweets entfernt, die beide Hashtags (#twitterlehrerzimmer und #twlz) enthielten oder von einem Computerprogramm (Bot; *Tw!tterlehrerzimmer*) automatisch retweetet worden waren. Der resultierende Ausgangsdatensatz (D1) bestand aus 128.422 Tweets von 21.328 Usern. Von diesen 21.328 Usern twitterten 9727 User (46 %) ausschließlich unter Verwendung des Hashtags #twitterlehrerzimmer, 4026 User (19 %) ausschließlich unter Verwendung des Hashtags #twlz und 7575 User (36 %) mindestens einen Tweet unter Verwendung beider Hashtags. Einerseits wird deutlich, dass ein großer Anteil der User unter beiden betrachteten Hashtags twitterte, wodurch die Prämisse einer gemeinsamen Community gestärkt wird. Andererseits folgen User bei Twitter keinen Hashtags sondern anderen Usern (in diesem Fall dem Bot *Tw!tterlehrerzimmer*), sodass die Informationen zu den verwendeten Hashtags in Tweets nur bedingt aussagekräftig für die Zugehörigkeit zu einer Community sein können.

Um die Tweets für die quantitativen Textanalysen der diskutierten Themen nutzen zu können (F1 und F2), wurden sehr kurze (weniger als drei Worte) und doppelte Tweets (Retweets) sowie URL-Adressen, Emojis und Emoticons innerhalb der Tweets entfernt sowie gängige Verfahren der Computerlinguistik angewandt. Aus den so aufbereiteten Tweets wurden alle 14.318 Tweets von 3237 Usern in den Analysen verwendet, die während der bundesweiten Schulschließungen (16. März–27. April 2020) veröffentlicht worden waren (Datensatz D2). Zusätzlich wurden alle 6731 Tweets von 1853 Usern verwendet, die vor den Schulschließungen (06. Januar bis 17. Februar 2020) veröffentlicht worden waren (Datensatz D3). Diese beiden Zeiträume wurden so ausgewählt, dass Themen vor und während der bundesweiten Schulschließungen für gleichlange Zeiträume verglichen werden konnten.

Um die schwerpunktmäßig thematisierten Chancen und Herausforderungen (F3) effizient im Rahmen einer qualitativen Inhaltsanalyse analysieren zu können (siehe Abschnitt *Statistische Analysen*), wurden diejenigen Tweets ausgewählt, die ein besonderes Echo im Twitter-Lehrerzimmer während der bundesweiten Schulschließungen auslösten, das heißt überdurchschnittlich viele Reaktionen hervorriefen. Dazu wurden aus dem Ausgangsdatensatz für den Zeitraum während der Schulschließungen, diejenigen Tweets ausgewählt, die besonders häufig weiterverbreitet wurden (viele Retweets), besonders starke Zustimmung erhalten haben (viele Likes) oder besonders intensiv diskutiert wurden (viele Comments). Es kann davon ausgegangen werden, dass durch diese Auswahlmechanismen diejenigen Tweets gefiltert wurden, die die Online-Community Twitter-Lehrerzimmer besonders beschäftigten. Insgesamt wurden 270 Tweets ausgewählt (Datensatz D4; für eine detaillierte Beschreibung des Filterprozesses siehe ebenfalls Anhang A1).

#### Variablen

Mit jedem Tweet sind Variablen auf Tweetebene (z. B. Anzahl der Likes, Anzahl der Retweets) und Userebene (z. B. Anzahl der Follower, Anmeldedatum bei Twitter) verknüpft. Bezüglich der Tweets wurden in dieser Studie der Inhalt (d. h. der veröffentlichte Text), das Datum der Veröffentlichung, die Anzahl der Likes, die Anzahl der Comments und die Anzahl der Retweets verwendet. Bezüglich der User, die die Tweets veröffentlicht haben, wurden die Anzahl der Follower (d. h. Anzahl der User, die einem bestimmten User folgen), Anzahl der Freunde (d. h. die Anzahl der Personen, denen ein User folgt) und das Anmeldedatum bei Twitter verwendet.

### Statistische Analysen

Zur Beantwortung der Forschungsfragen wurde ein Mixed-Methods-Analyseansatz verwendet (Johnson et al. 2007), um sowohl die Breite der Themen erfassen als auch ein tieferes Verständnis wahrgenommener Chancen und Herausforderungen während der Schulschließungen herausarbeiten zu können (Triangulation: Denzin 2012). Alle quantitativen Textanalysen wurden mit R berechnet (v4.0.2; R Core Team 2020) unter Verwendung von RStudio (v. 1.3.959; RStudio Team 2020). Die qualitativen Textanalysen (Inhaltsanalysen) wurden mit der Software MAXQDA (Version 20.0.5) durchgeführt.

Um die Themen zu identifizieren, die für die Online-Community Twitter-Lehrerzimmer während der bundesweiten Schulschließungen besonders drängend waren (F1), wurden zunächst über die einzelnen Tweets während der Schulschließungen hinweg die Substantive, als Indikatoren von Themen, automatisch mit der Textmarkierungsfunktion *udpipe_annotate* des R Paketes *udpipe* (v0.8.3; Wijffels 2019) markiert (*Parts of Speech tagging*). Dabei wurden ebenfalls verwendete Hashtags als Substantive berücksichtigt und Substantive auch mehrmals gezählt, wenn sie mehrmals in einem Tweet vorkamen. Anschließend wurden die absoluten Auftretenshäufigkeiten der Substantive als Indikator dafür verwendet, wie drängend ein Thema war. Ein Thema wurde als umso drängender eingestuft, je häufiger dieses erwähnt wurde.

Zusätzlich wurden Bigrams auf Basis aller Worte analysiert. In Bigrams werden zwei direkt aufeinander folgende Worte betrachtet, um die Beziehung von Worten untereinander und Zusammenhänge zwischen Themen deskriptiv zu beschreiben (Bekkerman und Allan 2003). In Bigram-Netzwerken werden diejenigen Worte sichtbar, die besonders häufig mit mehreren anderen Worten verknüpft sind (Knotenpunkte) und die somit ein Anhaltspunkt für Themenfelder (d. h. zugehörige Inhalte) bieten. Es wurden die Netzwerke derjenigen Bigrams betrachtet, die mindestens 10-Mal (vor den Schulschließungen) beziehungsweise 17-Mal (vor und während der Schulschließungen) auftraten. Die cut-off Werte wurden so gewählt, dass die Netzwerke übersichtlich und interpretierbar blieben, also sowohl eine manuelle Auswertung als auch eine übersichtliche grafische Darstellung der Themenfelder möglich war. Explorative Analysen unterschiedlicher Schwellenwerte zeigten, dass sich die zentralen Themenfelder durch niedrigere als die final gewählten Schwellen kaum beeinflussten, sondern lediglich alleinstehende Wortpaare ergänzt wurden, die nicht mit den Netzwerken verbunden und somit wenig aussagekräftig waren.

Um die Unterschiede der Themen im Twitter-Lehrerzimmer vor und während der bundesweiten Schulschließungen (F2) zu analysieren, untersuchten wir, welche Substantive und Bigrams besonders charakteristisch für die jeweiligen Betrachtungszeiträume waren. Dafür nutzten wir klassische computerlinguistische tf-idf-Analysen (*term frequency-inverse document frequency*) von Substantiven und Bigrams in Tweets vor und während der Schulschließung (Silge und Robinson 2017). Um die inhaltliche Bedeutung der Substantive beschreiben zu können, wurden Korrelationen der jeweils drei charakteristischsten Substantive mit allen anderen in den Tweets verwendeten Worten berechnet. Es wurden die zehn Worte mit den jeweils stärksten Zusammenhängen, die zugleich bezüglich der Forschungsfrage inhaltlich relevant waren, betrachtet.

Um einen möglichst gegenstandsnahen Einblick in die schwerpunktmäßig thematisierten Chancen und Herausforderungen während der bundesweiten Schulschließungen zu erhalten (F3), führten wir eine qualitative Inhaltsanalyse derjenigen 270 Tweets durch, die ein besonderes Echo in der Twitter-Community erzeugt hatten (siehe Anhang A1 für eine Beschreibung des Filterprozesses). Bei der Analyse folgten wir dem Prozessmodell induktiver Kategorienbildung nach Mayring (2015). In einem ersten Schritt wurde für alle 270 Tweets die Analyseeinheiten bestimmt. Als Analyseeinheiten legten wir die Tweetebene sowohl als Kodier- als auch als Kontexteinheit fest. In einem zweiten Schritt wurde jeder Tweet paraphrasiert, indem die Inhalte der Tweets auf zentrale Stichworte reduziert wurden. Teilweise waren in Tweets Dokumente verknüpft (z. B. Videos, Blogeinträge) und ohne diese inhaltlich nicht interpretierbar. Daher wurden alle verlinkten Dokumente ebenfalls separat zum Tweet paraphrasiert. In einem dritten Schritt wurden die Paraphrasen entsprechend der zentralen Inhalte, die sich unter den Oberkategorien *Chancen* und *Herausforderungen* subsumieren ließen, zu Subkategorien von Chancen und Herausforderungen zugeordnet. Dabei wurden zugleich bedeutungslose Phrasen entfernt sowie Phrasen gleichen Bedeutungsinhaltes zusammengefasst. In einem vierten Schritt wurde das finale Kategoriensystem zu Chancen und Herausforderungen basierend auf den reduzierten Subkategorien entwickelt. Diese Schritte wurden zunächst von einem Rater für 81 der 270 Tweets (30 %) durchgeführt. Anschließend wurde das Kategoriensystem basierend auf denselben 81 Tweets gemeinsam mit einem weiteren Rater überarbeitet, woraus 23 Kategorien resultierten. Beide Rater werteten anschließend unabhängig voneinander weitere 75 Tweets (28 %) aus mit zufriedenstellender Übereinstimmung (70 %). Unterschiede in den Ratings wurden für das finale Kategoriensystem konsensvalidiert (Anhang A2, Tab. 2). Schließlich kodierte ein Rater alle 270 Tweets, wobei Mehrfachkodierungen zulässig waren.

Die anschließende Analyse der identifizierten Chancen und Herausforderungen umfasste drei Schritte: Zuerst wurde durch Häufigkeitsanalysen bestimmt, wie drängend die Chancen und Herausforderungen waren. Die Chancen und Herausforderungen wurden als umso drängender eingestuft, je häufiger sie thematisiert wurden. Anschließend wurde das Echo von Chancen und Herausforderungen im Twitter-Lehrerzimmer durch prozentuale Anteile an Tweets analysiert, die besonders häufig verbreitet, denen besonders stark zugestimmt und die besonders intensiv diskutiert worden waren. Dafür wurden Tweets ausgewählt, deren Anzahl an Retweets, Likes oder Comments jeweils mindestens den Mittelwerten über alle 270 Tweets entsprachen. So wurden 87 Tweets mit mindestens 31 Retweets, 81 Tweets mit mindestens 131 Likes und 86 Tweets mit mindestens 13 Comments ausgewählt. Zuletzt wurden die Beziehungen der Chancen und Herausforderungen durch die Analyse der Häufigkeiten gemeinsamen Auftretens in den Tweets bestimmt.

## Ergebnisse und Diskussion

### Beschreibung des Twitter-Lehrerzimmers

Um zunächst einen Überblick über das Twitter-Lehrerzimmer zu erhalten, wurden zentrale Merkmale der User und der abgesetzten Tweets im gesamten Ausgangsdatensatz (d. h. alle 128.422 Tweets von 21.328 Usern im Zeitraum 01. November 2019 bis 03. Juni 2020; Datensatz D1) untersucht. Die User, die in dem vorliegenden Zeitraum Beiträge in der Twitter-Community veröffentlichten, hatten sich zwischen dem 21. Juli 2006 und dem 01. Juni 2020 bei Twitter angemeldet (*SD* = 3,74 Jahre). Die Anmeldezahlen der letzten vier Jahre legen einen stetigen Zuwachs des Twitter-Lehrerzimmers nahe. Vergleicht man die Anmeldungen der Monate März bis April aus 2020 mit den entsprechenden Anmeldungen aus den Vorjahren, so sind die Anmeldezahlen im Jahr 2020 höher (Beispiel März: 2018: *n* = 193; 2019: *n* = 248; 2020: *n* = 410). Für Mai 2020 zeigte sich der niedrigste Wert an Anmeldungen seit dem Jahr 2008. Insgesamt legen diese Befunde nahe, dass die Corona-Pandemie mit einem Zuwachs der Anmeldungen bei Twitter und einer verstärkten Aktivität der Lehrpersonen im Twitter-Lehrerzimmer einherging, während die Kurve im Mai bereits wieder abflachte.

**Figure 2:**
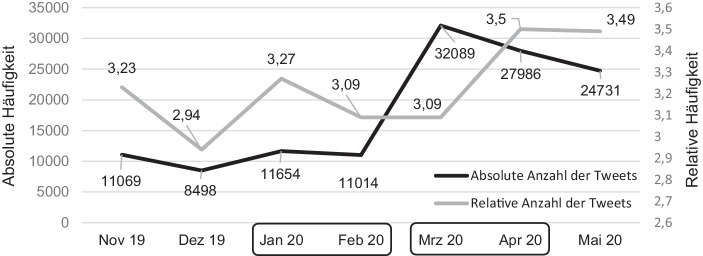

Um die Aktivität im Twitter-Lehrerzimmer vor und während der Schulschließungen abzubilden, wurden die absoluten Häufigkeiten der Tweets des gesamten Ausgangsdatensatzes pro Monat und die relativen Häufigkeiten der Tweets pro User pro Monat analysiert (Abb. 2). Zwischen Februar und März 2020 fällt ein deutlicher Anstieg der absoluten Häufigkeiten und zwischen März und April 2020 ein Anstieg der relativen Häufigkeiten an veröffentlichten Tweets auf. Diese Anstiege untermauern die Annahmen, dass das Twitter-Lehrerzimmer besonders zu Beginn der Schulschließungen einen Zulauf an Usern verzeichnete und während der Schulschließungen intensiver von Usern genutzt wurde.

Bei den 44.040 veröffentlichten Tweets des Ausgangsdatensatzes D1 der 11.304 User während der Schulschließungen, fällt auf, dass die meisten Tweets nicht retweetet (68 %), geliket (73 %) oder kommentiert (85 %) wurden. Das bedeutet, dass die meisten Tweets für sich stehen, ohne dass auf diese von anderen Usern reagiert wurde. Darüber hinaus zeichnen sich User, die sich während der Schulschließungen aktiv im Twitter-Lehrerzimmer beteiligten, durch eine große Heterogenität bezüglich ihrer Vernetztheit aus (Follower: *M* = 1463,00; *SD* = 22.458,73; Freunde: *M* = 690,20; *SD* = 2457,26).

### Themen (bezüglich digitalen Unterrichts) im Twitter-Lehrerzimmer während der bundesweiten Schulschließungen (F1)

**Figure 3:**
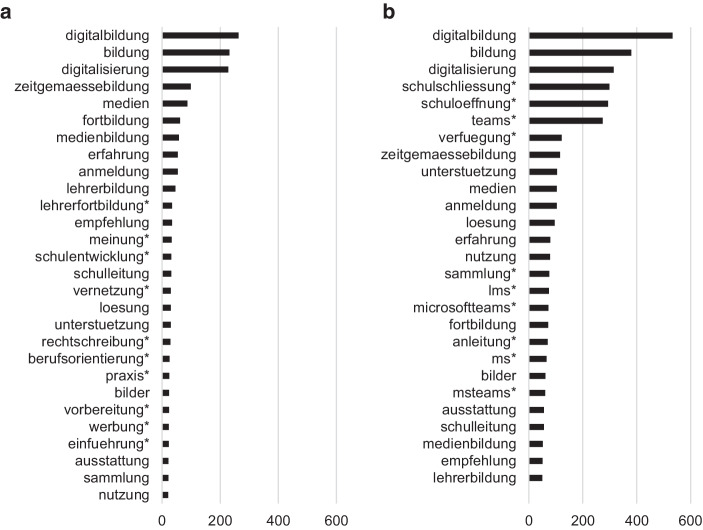

Abb. 3b zeigt die häufigsten genannten Substantive im Twitter-Lehrerzimmer während der Schulschließungen. Es wird deutlich, dass die Themen *Digitale Bildung* (z. B. repräsentiert durch *Digitalbildung, Digitalisierung, Bildung*), *Gegenseitige Hilfe* (z. B. repräsentiert durch *Anleitung, Empfehlung, Erfahrung, Lösung, Unterstützung*) oder *Konkrete Tools zum Lehren und Lernen* (z. B. repräsentiert durch Microsoft Teams [*ms, teams, msteams, microsoftteams*], Lernmanagementsystem [*lms*]) dominant sind. Zieht man zusätzlich die häufigsten Bigrams heran, so verfestigt sich dieser Eindruck: So wurden zum Beispiel die Worte *digital* bzw. *online* besonders häufig zusammen mit dem Wort *Unterricht* genannt (144 Nennungen). Ebenfalls traten die Wortkombinationen *teachfromhome* und *googleedu* (58 Nennungen), *MS Teams* (47 Nennungen), *tolle Idee* (43 Nennungen), sowie das Wort *digital* mit den Worten M*edien* (47 Nennungen), *Bildung* (41 Nennungen) und *Tools* (37 Nennungen) häufig gemeinsam auf.

**Figure 4:**
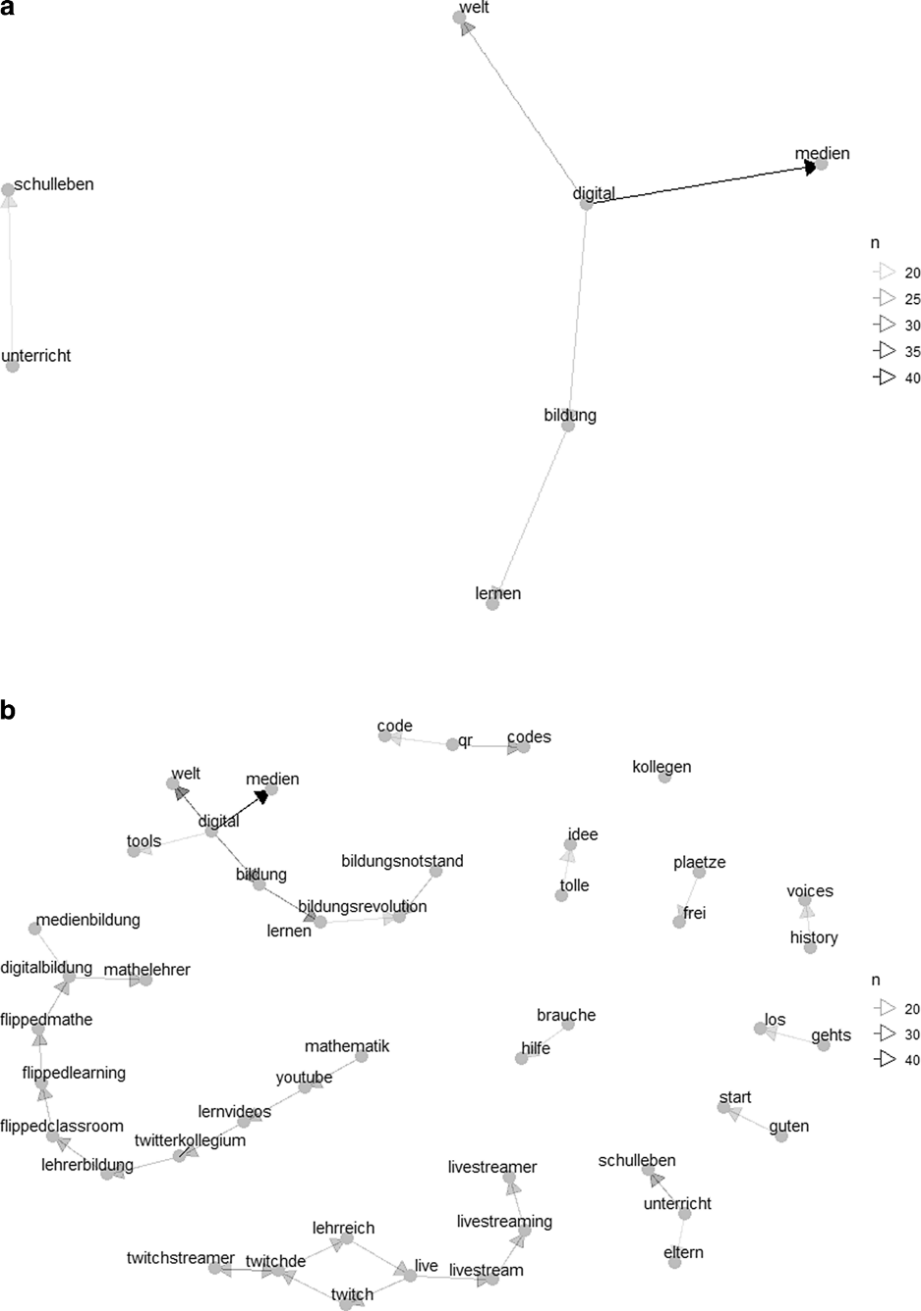

Des Weiteren lassen sich im Bigram-Netzwerk (Abb. 4c) drei größere Netzwerke identifizieren, die als Themenbereiche interpretiert werden können: Erstens wurden die prominentesten Knotenpunkte *digital* und *Lernen*, welche auch selbst direkt miteinander verknüpft sind, häufig genannt mit Worten wie *Unterricht, Bildung, Medien, online, Lehren* und *Klassenzimmer*. Die Größe des Netzwerkes deutet darauf hin, dass dieser Themenbereich unter Berücksichtigung vielfältiger Gesichtspunkte diskutiert wurde. Zudem lässt die hohe Sättigung der Pfeile zwischen einzeln Worten auf eine intensive Diskussion schließen. Ebenfalls im Netzwerk enthalten ist der Begriff *Fernunterricht*, der spezifisch für die Zeiten der Schulschließung sein dürfte. Zweitens stehen in einem weiteren Netzwerk synchrone Online-Angebote im Fokus: dieses Netzwerk ist über Begriffe wie *Livestreaming*/*Livestream* an den ersten Themenbereich angebunden, bildet jedoch einen eigenen Fortsatz. Dieser Fortsatz ist durch die Nennung des live-streaming-Videoportals *Twitch* (https://twitch.tv) dominiert, welches eigentlich vorrangig zum Übertragen von Videospielen Anwendung findet. Es ist anzunehmen, dass Lehrpersonen dieses Portal für das Übertragen von Unterrichtseinheiten in Betracht zogen oder über eine solche Nutzung berichteten. Drittens lässt sich ein Netzwerk erkennen, welches asynchrone Lerngelegenheiten thematisiert. In diesem Themenbereich steht die Methode *FlippedClassroom*/*FlippedLearning* im Mittelpunkt – eine Methode, welche vorsieht, dass der Wissenserwerb und die Stoffvermittlung im Selbststudium stattfinden, während deren Anwendung im Unterricht geschieht. Auch hier wurden mit *HomeSchooling* und *HomeOffice* Worte genannt, die insbesondere im Zuge der Schulschließungen aufgetreten sein dürften und den Schluss nahelegen, dass diese Unterrichtsmethode als Möglichkeit zur asynchronen Aufbereitung von Lernmaterial diskutiert worden ist. Dazu wurden einerseits Worte genannt, die auf einen Austausch innerhalb der Berufsgruppe schließen lassen (*Lehrerkollegium, Twitterkollegium*) sowie Beispiele für die Möglichkeit zur asynchronen Bereitstellung von Lerneinheiten (z. B. die Videoplattform *YouTube *[https://youtube.com]). Hervorzuheben ist, dass in diesem Netzwerk explizit der Fachbereich Mathematik zu finden ist (*FlippedMathe, Mathematik, Mathelehrer*). Schließlich gibt es eine Reihe von kleinen, isolierten Netzwerken. Vernachlässigt man bei diesen häufige Wortfolgen der natürlichen Sprache (z. B. *herzlichen Dank, nächste/letzte Woche, aktuelle Situation*) und Wortfolgen, die für konkrete Anwendungen stehen (z. B. *Microsoft Teams, Google Meet, Office 365*) bleiben Wortkombinationen übrig, die auf den Austausch von Erfahrungen abzielen (z. B. *brauche Hilfe, Verfügung stellen, Fragen stellen, Tipps* [und] *Tricks, tolle*/*gute Idee*). Insgesamt lassen diese großen und kleinen Netzwerke darauf schließen, dass sich Lehrpersonen im Twitter-Lehrerzimmer während der Schulschließungen über verschiedenste Möglichkeiten zur Umsetzung digitalen Unterrichts austauschten.

**Figure 5:**
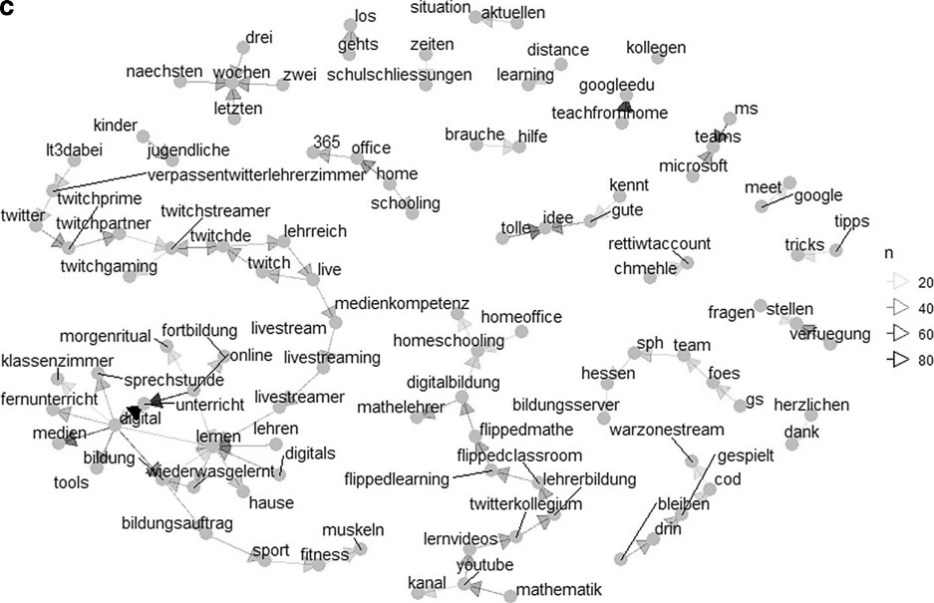

### Unterschiede der Themen im Twitter-Lehrerzimmer vor und während der bundesweiten Schulschließungen (F2)

Analog zur Forschungsfrage F1 wurden zunächst die häufig genannten Substantive betrachtet (über 50-Mal genannt) und für Tweets vor und während der Schulschließungen verglichen. Es zeigte sich, dass die drei häufigsten Substantive (*Digitalbildung, Bildung, Digitalisierung*) vor und während der Schulschließungen die gleichen waren und sogar in ihrer Reihenfolge erhalten blieben (Abb. 3).

Das Thema *Digitale Bildung* schien somit über die Zeit hinweg stabil aufzutreten und die Community Twitter-Lehrerzimmer zu beschäftigen. Weiter überschritten alle Substantive, die vor den Schulschließungen mehr als 50-Mal genannt wurden auch während der Schulschließungen diese Häufigkeitsschwelle. Dies kann als Hinweis darauf gedeutet werden, dass die Themen, die vor den Schulschließungen drängend waren, auch während der Schulschließungen drängend blieben. Allerdings überschritten vor den Schulschließungen deutlich weniger Worte das Häufigkeitskriterium, sodass während der Schulschließungen neue Worte hinzukamen. Das liegt auch darin begründet, dass vor den Schulschließungen insgesamt weniger Tweets abgesetzt wurden. Um die geringere Anzahl an Tweets vor den Schulschließungen zu berücksichtigen, wurden für diesen Zeitraum zusätzlich diejenigen Substantive betrachtet, die mindestens 20-Mal genannt wurden (Abb. 3a). Auch unter Berücksichtigung des niedrigeren Kriteriums tauchten vor den Schulschließungen Substantive wie *Verfügung* (im Sinne von „zur Verfügung stellen“), *Anleitung, Microsoft Teams* oder *lms *(Lern-Management-System) jedoch nicht unter den häufigsten Substantiven auf. Dass diese Worte während der Schulschließungen häufig genannt wurden, lässt darauf schließen, dass in diesem Zeitraum ein hohes Interesse am Austausch über Erfahrungen, Materialien und insbesondere Expertise bezüglich spezifischer Software oder Tools, im Twitter-Lehrerzimmer aufkam. Auch bei der Betrachtung von Bigrams zeigte sich, dass bereits vor den Schulschließungen das Thema Digitalisierung diskutiert worden war, was an der gemeinsamen Nennung der Worte *Digital Medien* (44 Nennungen), *digital Welt* (24 Nennungen), *digital Bildung* (21 Nennungen), *QR Codes* (17 Nennungen) und *Digitalbildung Mathelehrer* (16 Nennungen) zu sehen ist. Vor den Schulschließungen waren jedoch auch allgemein zu fassendere Wortkombinationen wie *Bildung Lernen* (21 Nennungen), *Unterricht Schulleben* (18 Nennungen) und *Bildungsrevolution Bildungsnotstand* (16 Nennungen) prominent vertreten. Es ist also anzunehmen, dass sich Lehrpersonen während der Schulschließungen stark mit digitalen Unterrichtsangeboten beschäftigten und der Fokus auf Themen der Digitalisierung im Twitter-Lehrerzimmer den hohen Bedarf an Erfahrungsaustuasch zu digitalen Lehr-Lern-Angeboten widerspiegelt.

Die Gegenüberstellung der Bigram-Netzwerke vor und während der Schulschließungen bei gleichbleibender Häufigkeit deren Auftretens (Abb. 4a, c) zeigt erneut die intensivere Aktivität im Twitter-Lehrerzimmer während der Schulschließungen. Auffällig ist wieder, dass die Worte *digital* und *Medien* bereits vor den Schulschließungen häufig gemeinsam mit den Worten *Bildung* und *Lernen* genannt wurden, was zum einen darauf schließen lässt, dass diese Themenfelder bereits vor den Schulschließungen drängend waren und zum anderen erneut zeigt, dass während der Schulschließungen weitere teils sehr spezifische Aspekte hinzukamen (z. B. *Fernunterricht*). Erneut wurde das Häufigkeitskriterium für den Zeitraum vor den Schulschließungen angepasst (*n* ≥ 10), um zu berücksichtigen, dass in diesem Zeitraum insgesamt weniger getwittert wurde (Abb. 4b). Die Netzwerke zeigen, dass sich die Themenfelder vor und während der Schulschließungen inhaltlich wenig unterschieden. Auch für den Zeitraum vor den Schulschließungen lassen sich drei zentrale Netzwerke differenzieren, welche – wie im Zeitraum während der Schulschließungen – die Themen Digitale Bildung (z. B. *digital, Medien, Bildung, Lernen*), synchrone Lerngelegenheiten (z. B. *Twitch, live, livestream*) und asynchrone Lerngelegenheiten (z. B. *YouTube, Lernvideos, FlippedClassroom*) beinhalten. Ebenfalls deuten kleinere Netzwerke (z. B. *brauche-Hilfe, tolle-Idee*) darauf hin, dass das Twitter-Lehrerzimmer schon vor den Schulschließungen für Austausch und Vernetzung hinsichtlich digitalen Unterrichts genutzt worden war.

Während erste Hinweise zu Unterschieden von Themen bereits durch den Abgleich der Auftretenshäufigkeit von Worten vor und während der Schulschließungen beschrieben wurden, bieten *tf-idf-Analysen* zusätzlich die Möglichkeit, charakteristische Worte und Bigrams für die beiden Beobachtungszeiträume zu identifizieren. Bezüglich der Substantive zeigte sich, dass der Zeitraum vor den Schulschließungen besonders durch die drei Worte *Lernzukunft20, Biko20* und *Bildungsnotstand* charakterisiert war. Die für den Zeitraum vor den Schulschließungen charakteristischen Substantive stammten aus Hashtags, die sich zum einen auf spezifische Bildungsevents (Lernen der Zukunft, Januar 2020, Frankfurt; Bildungskongress 2020, Februar 2020, Köln) und zum anderen auf Ausführungen zur Situation und Gestaltung gelingender Bildungsprozesse beziehen. Zieht man für die Interpretation der drei Substantive diejenigen Worte hinzu, die am stärksten mit diesen drei Substantiven korrelierten und zugleich für die Forschungsfrage inhaltlich relevant waren, so ergibt sich ein recht homogenes Bild. So korrelierten die Worte *Lernzukunft20* und *Biko20 *am stärksten mit eher messespezifischen Worten (z. B. *Frankfurt, Werbung, Informationen, Wochenende*), während das Wort *Bildungsnotstand* am stärksten mit Worten wie *Bildungsrevolution, Bildung, Lernen* oder *Noten* zusammenhing. So zeigt sich zum einen, dass unterschiedliche Aspekte des Lehrens und Lernens (Lernen und Leistungsbeurteilung) thematisiert wurden und zum anderen, dass häufig der Wunsch nach Veränderung (Revolution) verknüpft wurde. Die tf-idf-Analysen der charakteristischen Bigrams (*Unterricht Schulleben, Bildungsnotstand Bildungsrevolution* und *Bildung Bildungsnotstand*) bekräftigen zudem, dass das Wort *Bildungsnotstand* eine zentrale Stellung für den Zeitraum vor den Schulschließungen einnahm. Zugleich wird sichtbar, dass der reguläre Unterricht und das Schulleben insgesamt thematisiert wurde. Diese thematische Breite ist während der Schulschließung so nicht auszumachen. So war der Beobachtungszeitraum während der Schulschließungen durch die Substantive *Homeschooling, Schulschließung* und *Schulöffnung* charakterisiert. Deutlich spiegeln sich in diesen Worten die einschneidenden Maßnahmen und damit einhergehenden Konsequenzen der Schulschließungen wider. Mit *Homeschooling* hingen am stärksten die Worte *Digitalbildung, Medienkompetenz* und *Experiment* zusammen. Diese Wortpalette bringt zum Ausdruck, dass der Fernunterricht und/oder das Homeschooling im Twitter-Lehrerzimmer als Phase der Ungewissheit wahrgenommen wurde, in welcher wahrscheinlich der Medienkompetenz der Akteure eine entscheidende Rolle zum Gelingen zugeschrieben wurde. Naheliegend ist, dass das Wort *Schulschließung* stark mit den Worten (Hashtags) *Schulmessenger, Remotelearning, Itscommunity* oder *Herausforderung* korrelierte. In diesen Worten werden zum einen Konsequenzen der Schulschließungen sichtbar, dass für einen einhergehenden digitalen Fernunterricht zum Beispiel Technologie bedeutsam ist, zum anderen aber zugleich, dass die Phase der Schulschließungen als Herausforderung empfunden wurde, die in einer Gemeinschaft gemeinsam durchlebt wird. Passend dazu sind die identifizierten Bigrams *Online Unterricht, Teachfromhome Googleedu* und *Twitter Twitchprime* charakteristisch für den Zeitraum während der Schulschließungen. Der digitale Unterricht und damit einhergehend digitale Tools standen im Fokus, während der reguläre Unterricht, das Schulleben insgesamt sowie spezifische Einzelevents wie Messen oder Kongresse (charakteristisch für den Zeitraum vor den Schulschließungen) während der Schulschließungen so gut wie keine Rolle spielten.

### Chancen und Herausforderungen im Twitter-Lehrerzimmer während der bundesweiten Schulschließungen (F3)

Die Tweets, die während der Schulschließungen das größte Echo erzeugten, wurden mit einer qualitativen Inhaltsanalyse klassifiziert. Einen Überblick über die 23 Kategorien und deren relativen Auftretenshäufigkeiten gibt Abb. 5. Diese 23 Kategorien lassen sich wiederum zu den übergeordneten Kategorien *Chancen* und *Herausforderungen* zuordnen. Es zeigte sich ein leichtes Übergewicht an Herausforderungen: In 198 der 270 Tweets (73 %) wurden Herausforderungen und in 166 Tweets (61 %) Chancen thematisiert.

**Figure 6:**
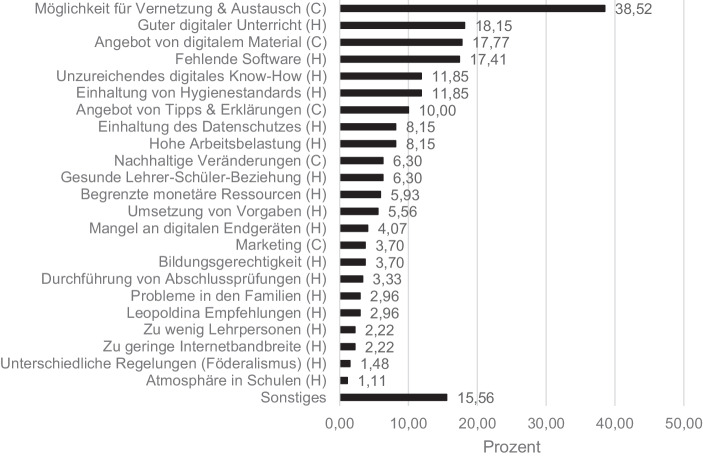

Zieht man die Auftretenshäufigkeit als Indikator dafür heran, wie drängend aktuelle Chancen und Herausforderungen sind, so sind die Herausforderungen der Gestaltung eines *guten digitalen Unterrichts* (49 Nennungen), die *fehlende Software* zum digitalen Lehren und Lernen (47 Nennungen) sowie *unzureichendes digitales Know-How* zur Durchführungen digitalen Unterrichts (32 Nennungen) die drei drängendsten Herausforderungen, die im Twitter-Lehrerzimmer formuliert worden sind. Als die drei größten Chancen können die *Möglichkeit für Vernetzung und Austausch* (104 Nennungen), das *Angebot von digitalem Material* (42 Nennungen) und das *Angebot von Tipps und Erklärungen* (27 Nennungen) gesehen werden. Insgesamt fällt auf, dass deutlich mehr Kategorien für Herausforderungen als Chancen abgebildet wurden. Dieser Unterschied kann verschiedene Ursachen haben. Zum einen könnten Herausforderungen tatsächlich differenzierter in den Tweets dargestellt worden sein, was die Vielfältigkeit unterschiedlicher Herausforderungen während der bundesweiten Schulschließungen widerspiegeln würde. Zum anderen könnte gleichermaßen ein Artefakt vorliegen, da Herausforderungen von den Twitter Usern meist explizit genannt worden sind, während die Chancen teilweise implizit angeführt und bei der Kodierung der Tweets als solche angenommen wurden. Daraus ergibt sich zugleich, dass die identifizierten Chancen auf zwei Analyseebenen zu interpretieren sind. Auf der ersten Analyseebene sind Chancen anzusiedeln, die im Twitter-Lehrerzimmer explizit benannt worden sind. Tweets, die solche Chancen enthielten, wurden unter der Kategorie *Nachhaltige Veränderungen* subsumiert und umfassten stets die Aussicht auf anhaltende Veränderungen bezüglich digitalisierten Unterrichts auch nach der Wiederöffnung der Schulen. Exemplarisch zeigt sich dies in folgendem Tweet: „Ein didaktischer Traum ist wahr geworden: Habe gestern für meine Schule eine Schullizenz für Padlet erworben. Viele Kolleg*innen möchten dauerhaft mit digitalen Pinnwänden arbeiten. #twitterlehrerzimmer“ (Marcus von Amsberg [@ivi_unterricht] 2020). In diesem Tweet wurden potenziell dauerhafte Veränderungsprozesse digitalen Unterrichts thematisiert, die vermutlich durch die besondere Situation der Schulschließungen initiiert wurden. Der „didaktische Traum“ bezieht sich dabei wohl nicht nur auf den Wunsch einer Lehrperson zu vermehrt digitalem Unterricht, sondern impliziert zugleich einen langwierigen Veränderungsprozess, in welchen nun endlich Schwung kommt. Diese Interpretation bekräftigt ein mit dem Tweet verlinktes Graphics Interchange Format (GIF) der Rede *I Have a Dream* von Martin Luther King Jr.

Auf der zweiten Analyseebene sind alle übrigen identifizierten Chancen (*Möglichkeit für Vernetzung und Austausch, Angebot von digitalem Material, Angebot von Tipps und Erklärungen, Marketing*) anzusiedeln. Diese wurden als Chancen anhand der Aussagen der analysierten Tweets angenommen und können zugleich als Reaktionen auf die Herausforderungen verstanden werden. So wurde zum Beispiel der Tweet „Ich freue mich, meine tolle Kollegin @dani_midd aus der #Grundschule hier im #twitterlehrerzimmer begrüßen zu dürfen. Sie freut sich über Tipps, wem sie im Bereich der #Grundschule folgen soll“ (grundschulmann [@grundschulmann] 2020), den Chancen *Möglichkeit für Vernetzung und Austausch* sowie *Angebot von digitalem Material* zugeordnet. Die so identifizierten Chancen eint, dass sie sich auf das Potenzial des Twitter-Lehrerzimmers als Community beziehen, die die Möglichkeit für Austausch und gegenseitige Unterstützung bietet. Des Weiteren können viele Herausforderungen gleichermaßen als Chance verstanden werden. Dass die Schulschließungen Lehrpersonen offensichtlich verstärkt in Diskussionen über die Realisierbarkeit digitalen Unterrichts (Herausforderung) führte (Wie sieht dieser aus? Welche Software gibt es? Ist der Datenschutz gewährleistet?), lässt sich ebenfalls als Potenzial für nachhaltige Veränderungen lesen.

Häufigkeitsanalysen der Kategorien können demnach abbilden, wie drängend Chancen und Herausforderungen sind. Es kann aber davon ausgegangen werden, dass bei derartigen Häufigkeitsanalysen nicht alle Chancen und Herausforderungen, die im Twitter-Lehrerzimmer ein großes Echo erzeugten, ausreichend berücksichtigt werden. Deutlich wird dies zum Beispiel in einem Tweet, der die in sehr wenigen Tweets angeführte Herausforderung der *Atomsphäre in Schulen* nach der Wiedereröffnung der Schulen thematisiert. Gleichwohl dieser Tweet mit 172 Retweets, 732 Likes und 35 Comments ein großes Echo im Twitter-Lehrerzimmer erzeugte, fällt dieser durch das Raster der Häufigkeitsanalysen. Aus diesen Gründen wurde die Auswertung der Chancen und Herausforderungen durch Analysen der Tweets mit den häufigsten Retweets, Likes und Comments angereichert. Zudem wurden Zusammenhänge von Chancen und Herausforderungen untereinander betrachtet.

### Chancen und Herausforderungen mit großem Echo im Twitter-Lehrerzimmer

In Tab. 1 ist ein Überblick der Kategorien dargestellt, die – gemessen an der Anzahl an Retweets, Likes und Comments – ein großes Echo in der Community Twitter-Lehrerzimmer erzeugten.

**Table 1:** 

Kategorie	Prozentualer Anteil an Tweets …		
*–*	… die häufig verbreitet wurden (*n* = 87) (in %)	… denen häufig zugestimmt wurde (*n* = 81) (in %)	… die intensiv diskutiert wurden (*n* = 86) (in %)
Angebot von digitalem Material (C)	**35**	15	14
Angebot von Tipps und Erklärungen (C)	**24**	–	–
Guter digitaler Unterricht (H)	19	**23**	18
Einhaltung von Hygienestandards (H)	13	**24**	12
Hohe Arbeitsbelastung (H)	–	13	**41**
Fehlende Software (H)	–	–	**20**
Unzureichendes digitales Know-How (H)	14	–	10
Nachhaltige Veränderungen (C)	12	12	–
Möglichkeit für Vernetzung und Austausch (C)	11	–	–

Zur Übersichtlichkeit sind nur Angaben mit einem prozentualen Anteil von mindestens 10 % abgetragen

Fett hervorgehoben sind die jeweils höchsten Anteile pro Kategorie

*C* Chance, *H* Herausforderung

Tweets mit *Angeboten von digitalen Materialien* sowie von *Tipps und Erklärungen* wurden am häufigsten in der Community weiterverbreitet, während Anfragen nach solchen eher kommentiert, jedoch kaum geteilt wurden (*Fehlende Software*). Dies kann erneut als Anzeichen für den großen Bedarf an der Bereitstellung von Materialien, Software(-hinweisen) sowie Tipps und Erklärungen zur Nutzung und Umsetzung im digitalen Unterricht gedeutet werden. Beispielsweise wurde ein Tweet mit dem Angebot zur kostenlosen Bereitstellung einer Geographie-App am häufigsten geteilt (711 Retweets). Weiter deutet die hohe Zahl an Kommentaren bei Tweets der Kategorie *hohe Arbeitsbelastung* darauf hin, dass die Umstellung auf (digitalen) Fernunterricht mit Mehrarbeit einherging oder diese zumindest stark diskutiert wurde. Tweets, die besonders viel Zustimmung erhielten (hohe Anzahl an Likes), beschäftigten sich mit den Themen *guten digitalen Unterrichts* und der *Einhaltung von Hygienestandards*. Interessant ist, dass diese Tweets meist auch geteilt und kommentiert wurden. Zusammenfassend lässt sich erkennen, dass Tweets, die als Chancen kategorisiert wurden, häufiger geteilt wurden, während als Herausforderung kategorisierte Tweets eher zu einer hohen Zahl an Likes und Kommentaren geführt haben, welche als empathische Reaktion oder emotionaler Support interpretiert werden könnten.

### Zusammenhang von Chancen und Herausforderungen

Um die Beziehungen von Chancen und Herausforderungen untereinander zu analysieren, wurden die gemeinsamen Auftretenshäufigkeiten der Kategorien in den 270 Tweets analysiert. Abb. 6 zeigt die identifizierten Verbindungsmuster zwischen den Kategorien.

**Figure 7:**
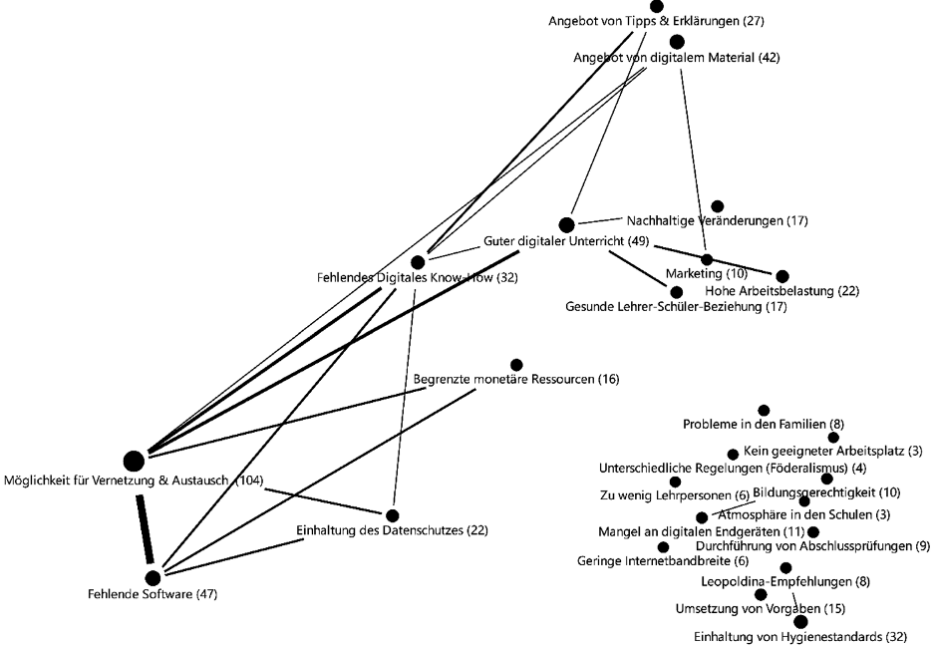

Drei bilaterale Beziehungen zwischen je zwei Aspekten traten besonders häufig zusammen in Tweets auf. Bei allen drei Beziehungen war die Chance *Möglichkeit für Vernetzung und Austausch* mit jeweils einer Herausforderung verknüpft. Am stärksten zeigte sich die Beziehung zu der Herausforderung *Fehlende Software*, wie im folgenden Beispiel: „Ich benötige euer Schwarmwissen: Apps für den Englischunterricht in der Grundschule!! Wer kann Tipps und Erfahrungsberichte geben! Gerne RT. Danke euch! [gefaltete Hände Emoji] #twlz #twitterlehrerzimmer #NRWedu #grundschule #englisch“ (Anna Fröhlich [@froehlichfresch] 2020). In dem Tweet wird eine dringliche Nachfrage (ausgedrückt durch zwei Ausrufezeichen) an Software für die Gestaltung von Unterricht deutlich. In anderen Tweets wurde zudem die fehlende Software zur Organisation und Durchführung von digitalem (Fern‑)Unterricht thematisiert. Häufig wurden die Nachfragen nach Software im Twitter-Lehrerzimmer mit Anforderungen an die Sicherstellung des Datenschutzes und einem geringen Finanzierungsaufwand kombiniert.

Die Unterstützung im Twitter-Lehrerzimmer wurde ebenfalls in Anspruch genommen, um über *unzureichendes digitales Know-How* zu berichten und/oder dieses zu kompensieren: „Ich als Lehrkraft habe das Gefühl, dass ich den ‚Profis‘ im Bereich Digitaler Unterricht gnadenlos hinterherlaufe. Viele meiner Kolleg*innen haben das Gefühl, dass sie mir als ‚Profi‘ gnadenlos hinterherlaufen. #twlz #twitterlehrerzimmer #digitalerunterricht #corona“ (Jens R. Jüttner [@jensrjuettner] 2020). Die Intention des Users lässt sich nur vermuten: Es könnte sein, dass mit diesem Tweet der Austausch mit Leidensgenossen im Twitter-Lehrerzimmer gesucht wurde. Zugleich könnte mit dem Tweet das Anliegen verbunden gewesen sein, auf die aktuelle, offensichtlich defizitäre Situation zum digitalen Know-How von Lehrpersonen aufmerksam zu machen. Außerdem wurde in Tweets häufig *fehlendes digitales Know-How* adressiert indem die Community zu einem konkreten Problem der Realisierung digitalen Unterrichts um Rat gefragt wurde (z. B. Fragen zu Funktionsweisen von Hard- oder Software).

Die dritte Verknüpfung der *Möglichkeit für Vernetzung und Austausch* zeigte sich mit der Herausforderung, *guten digitalen Unterricht* umzusetzen. Im Twitter-Lehrerzimmer wurden viele Fragen zu möglichen Kriterien aber auch zur Gestaltung guten (digitalen) (Fern‑)Unterrichts gestellt sowie Ideen und Ansätze guten Unterrichts diskutiert. Dies ist exemplarisch in folgendem Tweet zu erkennen: „Guten Morgen, liebes #twitterlehrerzimmer! Gibt es eine Übersicht mit Kriterien für sinnvolle und gute HomeSchooling-Angebote? [denkendes Gesicht Emoji]“ (Rebecca [@profesora_2903] 2020). In dem Tweet wird die Nachfrage nach Unterstützung von der Community bezüglich der Umsetzung guten (digitalen) (Fern‑)Unterrichts deutlich. In anderen Tweets ging es auch um Fragen, wie digitaler (Fern‑)Unterricht gestaltet sein sollte, ob beispielsweise das alleinige Verschicken von Übungsaufgaben als guter digitaler (Fern‑)Unterricht verstanden werden kann. In einigen Tweets wurden diese Fragen explizit mit der Chance nachhaltiger Veränderungen verknüpft, indem der Impuls in die Community gegeben wurde, über notwendige Veränderungen für einen guten digitalen Unterricht zu reflektieren, auch über die Phase der Corona-Pandemie hinaus.

Bezüglich der Herausforderung der Gestaltung *guten digitalen Unterrichts* fiel bei der Analyse der Tweets neben den drei skizzierten häufigsten bilateralen Verknüpfungen ein weiteres wiederkehrendes Muster von Herausforderungen auf: Herausforderungen wurden während der Schulschließungen häufig gegeneinander abgewogen. Sichtbar zum Beispiel in folgendem Tweet: „Wichtige Erfahrung aus den ersten Tagen Unterricht im Homeschooling Homeoffice: Weniger ist mehr! Erstmal mit den SuS gemeinsam lernen, wie online kommuniziert wird. Nicht mit Inhalten zuschießen. Das Wichtigste ist, dass wir LuL helfen, dass es den SuS jetzt gut geht. #twlz“ (Jens Schopper [@je_schop] 2020). Besonders fiel auf, dass die Herausforderung, *guten digitalen Unterricht* umzusetzen, in vielen Tweets ins Verhältnis zu der Herausforderung einer *gesunden Lehrer-Schüler-Beziehung* gesetzt wurde. In derartigen Tweets wurde betont, dass beispielsweise das Bereitstellen von Aufgaben in der Phase der Schulschließungen weniger relevant sei als den Kontakt zu den Schülerinnen und Schülern zu halten. Das könnte zum einen durch die Beziehung zur hohen Arbeitsbelastung erklärt werden, weil nicht allen Herausforderungen mit gleichmäßigem Engagement begegnet werden konnte und man deshalb Prioritäten setzen musste. Zum anderen könnten diese Tweets unterschiedliche Gewichtungen der Komponenten guten digitalen (Fern‑)Unterrichts andeuten.

### Zusammenfassung und Limitationen

Zusammenfassend kann festgehalten werden, dass die durch die Corona-Pandemie notwendigen Schulschließungen Lehrpersonen dazu drängten, ihren Unterricht in digitaler Weise zu gestalten. Die Analysen der Kommunikation im Twitter-Lehrerzimmer zeigten, dass das Thema digitaler Unterricht bereits vorher in dieser Online-Community diskutiert worden war, in der Ausnahmesituation jedoch stärker in den Fokus rückte. Erwartungsgemäß stieg die Anzahl der Tweets im Twitter-Lehrerzimmer an. Dabei decken sich die drei drängendsten Herausforderungen (*guter digitaler Unterricht, fehlende Software, unzureichendes digitales Know-How*) mit Befunden aus vorherigen Studien (Eickelmann et al. 2019; Vodafone Stiftung Deutschland 2020). Obwohl die Digitalisierung zuletzt viel Aufmerksamkeit und durch den Digitalpakt auch finanzielle Mittel erhielt, waren diese Themen ebenfalls zu erwarten, da die digitale Ausstattung der Schulen und die Vermittlung entsprechender Kompetenzen an die Lehrpersonen immer noch eine Aufgabe für die Bildungspolitik darstellen (Scheiter und Lachner 2019). Es bleibt zu hoffen, dass positive Erfahrungen, die im Zuge der Corona-Pandemie und dem damit verbundenen Fernunterricht mit digitalen Medien gesammelt wurden, nachhaltige Veränderungsprozesse für die Digitalisierung der Schulen anstoßen. Die intensivere Auseinandersetzung in Kombination mit der Notwendigkeit, digitalen Unterricht umzusetzen, könnte somit helfen, auf Seiten der Lehrpersonen Berührungsängste abzubauen und zugleich die Akzeptanz für digitalen Unterricht sowie digitale Fertigkeiten und Fähigkeiten zu fördern. Die Bildungspolitik könnte solche Plattformen nutzen, um einen Eindruck drängender Themen zu bekommen und die Frage zu beantworten, was aktuell von Lehrpersonen (auch über digitalisierungsbezogene oder spezifisch für den Fernunterricht relevante Themen hinaus) gebraucht und gefordert wird. Entsprechend wäre auch denkbar, dass dort Angebote und Informationen gezielt von bildungspolitischen Akteuren platziert werden. Es ist anzunehmen, dass soziale Medienplattformen wie Twitter unterstützend wirken können, Berührungsängste abzubauen, indem eine niedrigschwellige Möglichkeit für informelle Lerngelegenheiten und Austausch unter Kolleginnen und Kollegen ermöglicht wird. Die Zunahme der User als auch die intensiveren Diskussionen in der Online-Community können zumindest als Hinweise für einen erhöhten Bedarf an informellem Lerngelegenheiten und Austausch während der bundesweiten Schulschließungen gedeutet werden. Vor diesem Hintergrund sind soziale Medienplattformen insgesamt auch für die Zeit nach der Corona-Pandemie als vielversprechende Ergänzung zu traditionellen, größtenteils formalen Fortbildungsätzen (z. B. Workshops) zu sehen (vgl. Bruguera et al. 2019; Trust et al. 2016).

Allerdings stellt die Community Twitter-Lehrerzimmer wahrscheinlich eine positiv selektierte Stichprobe von Lehrpersonen dar, die sich durch eine hohe Medienaffinität auszeichnen dürfte. Es ist anzunehmen, dass sogenannte „Medienenthusiasten“ von ihren Erfahrungen aus dem digital gestützten Unterricht profitieren konnten. Jedoch stellte auch für diese Lehrpersonen der digitale Fernunterricht eine neue Situation dar. Zugleich ist davon auszugehen, dass die Einschränkung auf medienaffine Lehrpersonen in Verbindung mit den Defiziten, die in dieser Studie identifiziert wurden, für eine noch defizitärere aktuelle Situation bezüglich der Digitalisierung an Schulen in Deutschland spricht.

Des Weiteren sollte bedacht werden, dass in Online-Communities unterschiedliche Typen von Personen bezüglich der Verbreitung von Informationen existieren (Cha et al. 2012). Das bedeutet, dass in der Community Twitter-Lehrerzimmer neben vielen durchschnittlichen Usern zum Beispiel auch sogenannte „Meinungsführer“ aus unterschiedlichen Motiven Informationen streuen (Cha et al. 2012; Jungnickel 2017), wodurch die Dominanz von Themen potenziell verzerrt worden sein könnte. Gleichzeitig kann die Community Twitter-Lehrerzimmer nicht vollständig durch die dort online gestellten Tweets abgebildet werden. Neben aktiven Usern, die Tweets verfassen, liken und retweeten, gibt es in Online-Communities viele User, die hauptsächlich Informationen rezipieren (lurking; Frumin et al. 2018) und die trotzdem als Mitglieder einer Online-Community verstanden werden (Edelmann 2013). Die eigentliche Community ist daher mutmaßlich noch viel größer als unser Datensatz sichtbar machen kann. Schließlich beteiligen sich im Twitter-Lehrerzimmer nicht ausschließlich Lehrpersonen, sondern beispielsweise auch Eltern und Erziehungsberechtigte, Vertreter und Vertreterinnen von Verlagen oder aus der Bildungsadministration (wenn auch in geringer Zahl). Zur Generalisierung der Ergebnisse ist somit weitere Forschung notwendig.

Auch sollen weitere Einschränkungen dieser Studie erwähnt werden, die aus dem gewählten methodischen Vorgehen resultieren. Es wurde eine begründete Auswahl von Hashtags für diese Studie vorgenommen, sodass die Ergebnisse ausschließlich für die definierte Online-Community, die durch die beiden Hashtags #twittlerlehrerzimmer und #twlz repräsentiert werden, gültig sein können. Es sei darauf hingewiesen, dass im Bildungskontext weitere bedeutsame Hashtags verwendet werden, die hier nicht eingeschlossen wurden. In zukünftiger Forschung könnte eine breiter angelegte Auswahl die vergleichende Untersuchung verschiedener Communities ermöglichen. Auch wurden die Schwellenwerte für die Auswertung der Bigrams explorativ und in einem Abwägungsprozess zwischen der Möglichkeit einer manuellen Identifizierung von Themenfeldern auf der einen und der Möglichkeit einer übersichtlichen Darstellung auf der anderen Seite festgelegt. Ein quantitatives Kalkül (z. B. Orientierung des Schwellenwertes an Anzahl von Tweets in einem Analysezeitraum) hätte hingegen eine höhere Standardisierung ermöglicht. Somit ist nicht auszuschließen, dass Themenfelder übersehen wurden. Allerdings haben sich die zentralen Themenfelder bei verschiedenen, niedrigeren Schwellen nicht geänderten und es kann davon ausgegangen werden, dass die wichtigsten Themenfelder entdeckt wurden, da diese auch bei höheren Schwellen sichtbar bleiben sollten.

Schließlich sei darauf hingewiesen, dass Sentiment-Analysen die Ergebnisse zu den diskutierten Themen um eine emotionale Komponente hätten erweitern können. Dabei hätten zusätzlich Emojis und Emoticons berücksichtigt werden können. So wären zum Beispiel interessant gewesen, welche Themen im Vergleich vor und während der bundesweiten Schulschließungen eher positiv und welche eher negativ diskutiert wurden. Dies ist eine vielversprechende Forschungsrichtung für zukünftige Studien.

### Fazit

Die bundesweiten Schulschließungen können als Brennglas für Defizite im Bildungssystem bezüglich des Digitalisierungsprozesses betrachtet werden und bieten die Chance, als Katalysator für nachhaltige Veränderungen genutzt werden zu können. Die Befunde dieser Studie verdeutlichen, dass insbesondere die Frage, was guter digitaler (Fern‑)Unterricht überhaupt ist, Lehrpersonen im Twitter-Lehrerzimmer beschäftigte. Zur Beantwortung waren und sind Lehrpersonen hier noch weitestgehend auf sich allein gestellt, um geeignete Methoden und Datenschutz-konforme Tools zu identifizieren sowie Material zu erstellen oder zu finden. Interessant ist zudem, dass ein Austausch vorwiegend über Tools stattfand, aber weniger in Bezug auf didaktische Verfahren, wobei das Primat des Pädagogischen wenig Berücksichtigung fand. Daher braucht es dringend generalisierbare Forschungsbefunde sowie curricular motivierte und empirisch geprüfte Unterrichtsmaterialien. Darüber hinaus sollte der Umgang mit digitalen Medien und deren didaktische Einbindung in den Unterricht in die Lehrerausbildung sowie entsprechende Maßnahmen in der Fort- und Weiterbildung aufgenommen werden.

## Anhang A

### Anhang A1. Beschreibung der Datenaufbereitung

Um die Tweetinhalte in den Analysen nutzen zu können, wurde der Rohdatensatz aufbereitet. In einem ersten Schritt wurden diejenigen 8166 Tweets, die sowohl zum Hashtag #twitterlehrerzimmer als auch #twlz geladen wurden, also doppelt im Rohdatensatz vorkamen, entfernt. Es handelt sich dabei um Tweets, die nur einmal abgesetzt wurden und lediglich aufgrund der gewählten data scraping Methode (d. h. separates Downloaden von Tweets für die beiden Hashtags #twitterlehrerzimmer und #twlz) doppelt im Rohdatensatz vorkamen. Anschließend wurden die 47.098 Tweets gelöscht, die von einem Computerprogramm (Bot Tw!tterlehrerzimmer; https://twitter.com/bot_twlehrerz?lang=de) abgesetzt wurden. Der Bot ist so konfiguriert, dass alle Tweets mit den Hashtags #twitterlehrerzimmer, #twlz und #twitterlz automatisch retweetet werden. Der resultierende Ausgangsdatensatz (D1) bestand aus 128.422 Tweets von 21.328 Usern.

#### Tweets vor und während der Schulschließungen für quantitative Analysen (F1 und F2)

Zunächst wurden diejenigen Tweets, die weniger als drei Worte umfassten und die somit weniger aussagekräftig gewesen sein dürften, aus dem Ausgangsdatensatz (D1) entfernt. Weil bei den Forschungsfragen F1 und F2 die Breite der Themen im Fokus stand und um das Gewicht von Worten und Wortkombinationen nicht künstlich zu verfälschen, wurden alle nicht-automatischen Retweets entfernt. Um die Tweets in quantitativen Textanalysen verwenden zu können, wurden die Tweets weiter bereinigt. Dafür wurden zum Beispiel URL-Adressen und Emoticons entfernt. Zudem wurden Worte (*Stopwords*) entfernt, die sehr häufig auftreten und in der Regel wenig Relevanz für die Erfassung des Tweetinhalts besitzen (z. B. „ist“, „ein/e“). Die Stopwords wurden durch die Vereinigung von Listen an deutschsprachigen Stopwords aus den R Paketen *stopwords* (v 2.0; Benoit et al. 2020) und *lsa* (v0.73.2; Wild 2020) sowie 115 manuell definierten Stopwords (z. B. „liebes“, einzelne Buchstaben, Zahlen) erzeugt. Insgesamt wurden 561 Stopwords berücksichtigt. Bei quantitativen Textanalysen führen Worte, die inhaltlich dasselbe bedeuten aber unterschiedlich geschrieben sind (z. B. „Lehrer“ und „Lehrkräfte“) zu Auswertungsschwierigkeiten, da zum Beispiel Häufigkeiten der Worte nicht korrekt ausgezählt werden können. Eine automatisierte Reduktion von Worten auf ihren Wortstammbaum (*Trunkierung*) ging mit einer schwierigeren inhaltlichen Interpretation einzelner Worte einher, weil zum Beispiel das Wort „Eltern“ zu „Elt“ geändert wurde. Deshalb wurden häufig verwendete und inhaltlich zusammengehörige Worte wie beispielsweise „Schulen“ und „Schule“ oder „Lehrer“ und „Lehrkräfte“ manuell zusammengefügt. Aus den so aufbereiteten Tweets wurden diejenigen 14.318 Tweets von 3237 Usern ausgewählt, die während der bundesweiten Schulschließungen (16. März bis 27. April 2020) veröffentlicht wurden (Datensatz D2). Um die in den Tweets behandelten Themen vor und während der bundesweiten Schulschließungen vergleichen zu können, wurden zusätzlich 6731 Tweets von 1853 Usern ausgewählt, die im Zeitraum vom 06. Januar bis 17. Februar 2020 veröffentlicht wurden (Datensatz D3). Die gewählten Zeiträume waren somit gleich lang (42 Tage).

#### Tweets mit Echo im Twitter-Lehrerzimmer während der Schulschließungen für qualitative Analysen (F3)

Um einen Datensatz derjenigen Tweets zu erhalten, die ein besonderes Echo im Twitter-Lehrerzimmer während der bundesweiten Schulschließungen auslösten, wurden aus dem Ausgangsdatensatz für den Zeitraum vom 16. März bis 27. April 2020 (*n* = 44.040 Tweets), die Tweets ausgewählt, die besonders häufig verbreitet wurden (viele Retweets), besonders starke Zustimmung erhalten haben (viele Likes) oder besonders intensiv diskutiert wurden (viele Comments). Betrachteten wir diejenigen Tweets, die jeweils am häufigsten retweetet, geliket und kommentiert wurden, so wurde deutlich, dass die drei Tweet-Eigenschaften (Anzahl an Retweets, Anzahl an Likes, Anzahl an Comments) keine disjunkten Eigenschaften waren. Wählten wir zum Beispiel die Tweets aus, die am meisten retweetet wurden, so waren dies auch diejenigen Tweets, die am meisten gelikt oder kommentiert wurden. Aus diesem Grund untersuchten wir zunächst, wie die drei Tweet-Eigenschaften zusammenhingen. Dafür betrachteten wir die 43.179 Tweets des Ausgangsdatensatzes (D1), die entweder mindestens einmal retweetet oder mindestens einmal geliket oder mindestens einmal kommentiert wurden. Es zeigte sich, dass die Schwelle dafür, auf einen Tweet zu reagieren, für das Retweeten am niedrigsten war. Denn von den 43.179 Tweets gab es lediglich 449 Tweets, die nicht retweetet aber geliket oder kommentiert wurden. Das bedeutet, dass Tweets, die geliket oder kommentiert wurden, in den meisten Fällen auch retweetet wurden. Andersherum wurden 7084 Tweets nicht geliket aber retweetet und 21.901 Tweets nicht kommentiert aber retweetet. Ein Tweet, der retweetet wurde, konnte also nicht losgelöst davon analysiert werden, dass dieser Tweet auch geliket oder kommentiert wurde beziehungsweise davon, dass eine hohe Anzahl an Retweets mit einer hohen Anzahl an Likes und Comments einherging. Daher haben wir uns dafür entschieden, die Tweets, die am meisten retweetet wurden, ohne Berücksichtigung der Anzahl an Likes und Comments auszuwählen. Um eine Auswertung im Rahmen einer qualitativen Inhaltsanalyse durch eine handhabbare Anzahl an Tweets zu ermöglichen, entschieden wir uns dazu, die 100 meist retweeteten Tweets zu wählen. Für die beiden übrigen Tweet-Eigenschaften (Anzahl an Likes und Anzahl an Comments) wählten wir – unabhängig von der Anzahl der Retweets – je 100 Tweets für die Kombinationsmöglichkeiten aus hoher und niedriger Ausprägung der Anzahl von Likes und Comments pro Tweet aus (d. h. 1. hohe Anzahl an Likes und hohe Anzahl an Comments, 2. Hohe Anzahl an Likes und niedrige Anzahl an Comments, 3. Niedrige Anzahl an Likes und hohe Anzahl an Comments). Die Kombination aus niedriger Ausprägung sowohl der Anzahl von Likes als auch der Anzahl an Comments pro Tweet wurde nicht berücksichtigt, da diese Tweets kein Echo im Twitter-Lehrerzimmer erzeugten. In einem ersten Schritt wurden Gewichte (reelle Zahlen) x_i_ bestimmt, für welche nahezu 100 Tweets gefiltert wurden, deren Anzahl an Likes und Comments jeweils größer oder gleich den Mittelwerten plus x_i_ Standardabweichungen waren (d. h. Tweets mit hoher Ausprägung der Anzahl von Likes und Comments). Es wurde x_i_ = 4,27 gesetzt, weil damit 101 Tweets gefiltert wurden, was zu der kleinstmöglichen Abweichung von der angestrebten Anzahl von 100 Tweets bedeutete. Im nächsten Schritt wurden aus den 427 Tweets, deren Anzahl an Comments 4,27 Standardabweichungen über dem Mittelwert lagen, die 100 Tweets ausgewählt, die am wenigsten Likes erhielten (d. h. Tweets mit hoher Ausprägung der Anzahl von Comments aber niedriger Ausprägung der Anzahl an Likes). Zudem wurden aus den 161 Tweets, deren Anzahl an Likes 4,27 Standardabweichungen über dem Mittelwert lagen, die 100 Tweets ausgewählt, die am wenigsten kommentiert wurden (d. h. Tweets mit hoher Ausprägung der Anzahl von Likes aber niedriger Ausprägung der Anzahl an Comments). Insgesamt lagen somit 401 Tweets vor. Da die drei Kategorien (Anzahl der Retweets, Anzahl der Likes, Anzahl der Comments) nicht disjunkt waren, schlossen wir in einem letzten Schritt 131 Duplikate aus. Insgesamt wurden somit 270 Tweets von 194 Usern ausgewählt. Die 270 Tweets wurden zwischen 1‑ und 711-Mal retweetet, zwischen 0‑ und 1613-Mal geliket und zwischen 0‑ und 57-Mal kommentiert.

### Anhang A2

**Table 2:**

Bezeichnung der Kategorien	Definition	Fiktives Ankerbeispiel	Kodierregeln
*Chancen*			
Möglichkeit für Vernetzung & Austausch	Das Twitter-Lehrerzimmer als Netzwerk	„Liebes #twitterlehrerzimmer, kennt ihr gute spanische Lektüre für meine 10. Klasse? Am besten wären E‑Books, meine SuS wollen digitalen Lesestoff. ☺ Das Angebot der Schulbuchverlage ist eher dünn …“	Support des Twitter-Lehrerzimmers wird explizit eingefordert, indem z. B. Fragen gestellt werden. Es werden Impulse zur Diskussion in das Twitter-Lehrerzimmer gegeben. Es wird explizit Feedback von der Community Twitter-Lehrerzimmer eingefordert
Angebot von digitalem Material	Angebot von Soft- und/oder Hardware und/oder digitalem Unterrichtsmaterial	„Klasse, ich habe eine tolle Software gefunden, um meinen Unterricht zu streamen. Da kann man Dinge einfach erklären, indem man einfach auf diese mit einem Pointer verweist. Genial! ☺ Hier der Link …“	Es werden Hinweise zur Verfügbarkeit von Soft- und/oder Hardware sowie digitalem Unterrichtsmaterial gegeben. Digitales Unterrichtsmaterial (z. B. Arbeitsblätter als PDF) werden direkt geteilt
Angebot von Tipps & Erklärungen	Erklärungen und Hilfestellungen im Umgang mit digitaler Soft-/und/oder Hardware	„Ich habe eine Schritt-für-Schritt Anleitung dazu erstellt, wie man schnell eine Cloud für das Lehren und Lernen einrichten kann. Hier der Link zu dem PDF, natürlich kostenlos.“	Es werden (Schritt-für-Schritt)Anleitungen z. B. zur Verwendung von Software geteilt, um digitalen (Fern‑)Unterricht durchführen zu können. Es werden Sammlungen von hilfreichen Links und/oder Tipps zur Durchführung digitalen (Fern‑)Unterrichts geteilt. Es wird direkt auf Bedürfnisse (z. B. Fragen zum Einsatz digitaler Tools) reagiert. Es werden Erklärvideos erstellt und geteilt
Nachhaltige Veränderung	Die Phase der Schulschließungen als Chance für nachhaltige Veränderungen	„Wenn ich mit meinen Kollegen an anderen Schulen spreche, dann sehen ich, dass die Coronakrise der Digitalisierung in unseren Schulen einen echten Schub geben kann. Fantastisch!“	Das Potenzial der Schulschließungen für nachhaltige Veränderungen der Gestaltung von Lehr- und Lernprozessen und/oder Schule und/oder (digitalen) Unterricht und/oder Digitalisierung in Deutschland und/oder stärkere digitale Vernetzung über die Phase der Schulschließungen hinaus wird thematisiert
Marketing	Gewinnorientierte Unternehmen teilen ihre Produkte	„Wir bieten euch eine tolle kostenlose Online-Fortbildung an. Meldet euch einfach in unserem Newsletter auf unserer Homepage an und erhaltet zukünftig Informationen zu tollen Events!“	Es werden digitales Material (z. B. Software) und/oder Unterstützung (z. B. Online-Fortbildungen) von Unternehmen angeboten, denen ein kommerzielles Interesse unterstellt werden kann, weil die Angebote zukünftig mit Kosten verbunden sind
*Herausforderungen*			
Guter digitaler Unterricht	Kriterien und Gelingensbedingungen guten digitalen (Fern‑)Unterrichts	„Guter digitaler Unterricht besteht nicht darin, den SuS möglichst viele PDFs via E‑Mail zu schicken. Es gehört mehr dazu!“	Das Gelingen eines guten digitalen (Fern‑)Unterrichts wird explizit oder implizit thematisiert. Kriterien eines guten digitalen (Fern‑)Unterrichts, werden explizit oder implizit thematisiert
Fehlende Software	Software zur Organisation und/oder Durchführung und/oder inhaltlichen Ausgestaltung digitalen (Fern‑)Unterrichts fehlt	„Liebes #twlz, hat hier jemand einen Tipp, für eine gute Lernapp für den Englischunterricht, die ich mit meinen SuS verwenden kann? Zudem würde ich mich gern mit meinen Kollegen austauschen. Auch dafür einen Tipp?“	Mangel an Software direkt zum Lehren und Lernen wird explizit oder implizit (bspw. durch Nachfragen in der Community) thematisiert. Mangel an Software zur Organisation und Durchführung digitalen (Fern‑)Unterrichts wird thematisiert
Unzureichendes digitales Know-How	Kein ausreichendes Wissen für Durchführung digitalen (Fern‑)Unterrichts	„Liebes #tweitterlehrerzimmer, meine KuK und ich scheitern daran, eine Videokonferenz mit unserer Software durchzuführen. Wie geht das? Danke für eure Tipps“	Explizit oder implizit (bspw. durch Nachfragen zu Erklärvideos, geteilte Anleitungen) werden unzureichende Fähigkeiten von Lehrpersonen für einen digitalen (Fern‑)Unterricht thematisiert
Einhaltung von Hygienestandards	Einhaltung von Hygienestandards bei Schulöffnungen	„Die Schulen wieder zu öffnen ist Quatsch. Zu volle Klassen, kein Abstand möglich. Da mach ich nicht mit! Ich denke auch an die Risikogruppen …“	Der Aufwand der Einhaltung von Hygienestandards in den Schulen nach den Schulöffnungen wird thematisiert. Die Einhaltung von Hygienestandards in den Schulen nach den Schulöffnungen wird kritisch thematisiert
Einhaltung des Datenschutzes	Fragen/Unsicherheiten bezüglich der Einhaltung des Datenschutzes	„Wer kennt ein datenschutzkonformes Programm zum Chatten, damit ich mit meinen SuS Kontakt halten kann? Gängige Software scheint dafür ja eher nicht geeignet, weil nicht DSGVO konform …“	Datenschutzfragen werden bei Soft- und/oder Hardware thematisiert. Der Aufwand der Einhaltung des Datenschutzes wird explizit oder implizit thematisiert
Hohe Arbeitsbelastung	Digitaler (Fern‑)Unterricht geht mit hoher Arbeitsbelastung einher	„In den letzten Tagen beantworte ich tausende von E‑Mails, meine SuS wollen viel Feedback … die Arbeit hat durch das Homeschooling stark zu- und nicht abgenommen“	Eine hohe Arbeitsbelastung von Eltern und/oder Schülerinnen und Schülern und/oder Lehrpersonen durch digitalen (Fern‑)Unterricht wird explizit oder implizit (bspw. durch die Bereitstellung von Entspannungslisten) thematisiert
Gesunde Lehrer-Schüler-Beziehung	Aufrechterhaltung des Kontaktes von Lehrpersonen zu Schülerinnen und Schülern beim digitalisierten (Fern‑)Unterricht	„Liebes #twlz, in der aktuellen Situation ist die Beziehungsarbeit umso wichtiger. Leute, auch, wenn es schwieriger ist, haltet Kontakt zu euren SuS“	Explizit oder implizit (bspw. durch Ermahnungen, Kontakt zu Schülerinnen und Schülern aufrechtzuerhalten) wird die Bedeutsamkeit der Beziehung/des Kontaktes zwischen Lehrpersonen und Schülerinnen und Schülern insbesondere in Zeiten digitalen (Fern‑)Unterrichts wird thematisiert
Begrenzte monetäre Ressourcen	Finanzielle Mittel zur Durchführung digitalen (Fern‑)Unterrichts sind knapp	„Welche Software verwendet ihr zum Bearbeiten von PDFs? Ich suche eine gute, kostenfreie Software. Meine Schule stellt diese nicht zu Verfügung und im Internet viele ich viele sehr teure Angebote …“	Begrenzte finanzielle Mittel zur Anschaffung von Soft- oder Hardware werden betont. Es wird Soft- oder Hardware angeboten, die kostenfrei ist, die gleichzeitig aber nicht von gewinnorientierten Unternehmen beworben wird (Kategorie Marketing) und die zukünftig zu keinen Kosten führt. Finanzierung von Soft- oder Hardware durch politische Akteure wird gefordert
Umsetzung von Vorgaben	Herausforderung, Vorgaben umzusetzen	„Die Schule gibt vor, dass wir Unterricht machen sollen. Aber wie? Digital scheint zu riskant wegen Datenschutz, persönlich ist verboten, Arbeitsblätter können auch nicht gedruckt werden, weil keine Drucker …“	Die Umsetzung der Vorgaben durch Politik und/oder Institutionen (z. B. Schulen) wird kritisiert bzw. die Machbarkeit in Frage gestellt
Mangel an digitalen Endgeräten	Mangel an digitalen Endgeräten existiert	„Kann mir mal jemand sagen, wie das gehen soll? Keine Rechner, keine Tablets, keine Ausstattung an den Schulen. So kann digitaler Unterricht nicht gelingen!“	Es wird explizit die mangelnde Ausstattung mit digitalen Endgeräten von Eltern und/oder Schülerinnen und Schülern und/oder Lehrpersonen angesprochen. Es wird implizit auf einen Mangel an digitalen Endgeräten hingewiesen (z. B. indem digitale Endgeräte zur Verfügung gestellt werden). Es wird Unterstützung für die Anschaffung von digitalen Endgeräten gefordert
Bildungsgerechtigkeit	Gefahr der Verschärfung von Bildungsungerechtigkeiten	„#bildungsungrechtigkeit Wenn die Kinder zu Hause keine vernünftige Ausstattung haben, dann wird das Homeschooling die eh schon benachteiligten Kinder stark benachteiligen“	Es wird explizit die potenzielle Verschärfung von Bildungsungerechtigkeit durch digitalen (Fern‑)Unterricht thematisiert. Initiativen zur Ausstattung von Schülerinnen und Schülern, die z. B. nicht über digitale Endgeräte verfügen
Durchführung von Abschlussprüfungen	Abschlussprüfungen nach Schulschließungen sind herausfordernd	„Hi #twitterlehrerzimmer, es ist unvernünftig die Abschlussprüfungen unter diesen Umständen durchzuführen. Was hatet ihr vom Durchschnittsabitur?“	Die Durchführung von Abschlussprüfungen nach der Wiedereröffnung von Schulen wird explizit oder implizit als Aufgabe formuliert, die nicht einfach zu erledigen ist
Probleme in den Familien	Schwierige familiäre Situation	„Ich habe einen Schüler in meiner Klasse, von dem ich im Chat erfahren habe, dass er zu Hause geschlagen wurde. Es ist so wichtig, dass ihr euch um eure SuS kümmert“	Schwierige Situationen in Familien (z. B. häusliche Gewalt, Vereinbarkeit von Beruf und Homeschooling/Betreuung von Kindern, Existenzängste) in Folge der Maßnahmen zum Infektionsschutz (u. a. Schulschließungen, Kurzarbeit) werden thematisiert
Leopoldina Empfehlungen	Kritik an den Empfehlungen von Leopoldina	„Liebes #twlz, ich finde die Leopoldina-Empfehlungen unmöglich. War jemand von denen schonmal in einer echten Schule?“	Empfehlungen, die die Deutsche Akademie der Naturforscher Leopoldina – Nationale Akademie der Wissenschaften (Leopoldina), veröffentlicht hat, werden kritisiert
Zu wenig Lehrpersonen	Zu wenig Lehrpersonen für reibungslosen Unterrichtsbetrieb vorhanden	„Hey KuK, wie haben eh schon zu wenig Lehrer. Ohne mehr Personal, weiß ich nicht, wie wir den Unterricht durchführen sollen, wenn die Risikogruppe auch noch wegfällt“	Explizit (z. B. zu wenig Lehrpersonen generell oder besonders durch Wegfall von Lehrpersonen, die zur Risikogruppe gehören) oder implizit (z. B. Beschreibung umständlicher Arbeitsweisen resultierend aus Mangel an Lehrpersonen) wird thematisiert, dass zu wenig Lehrer für einen reibungslosen Unterrichtsbetrieb (insbesondere nach Wiedereröffnung der Schulen) eingestellt sind
Zu geringe Internetbandbreite	Instabile oder kaum verfügbare Internetverbindung	„Liebes #twitterlehrerzimmer, wie geht es euch? Könnt ihr Fernunterricht machen? Ich nicht, mein Internet versagt ständig.“	Die Instabilität von Anwendungen (insbesondere bei größeren Anwenderzahlen) wird thematisiert. Die Instabilität von Internetverbindungen in Schulen und/oder zu Hause wird thematisiert
Unterschiedliche Regelungen (Föderalismus)	Konfusion durch unterschiedliche Regelungen	„Ich bin verwirrt! Welche Regelungen gelten denn nun in welchem Bundesland? Ich komme nicht mehr hinterher …“	Es wird zum Ausdruck gebracht, dass Vorgaben (z. B. bezüglich Wiedereröffnungen von Schulen, Beschulung von Abschlussklassen) nicht über Bundesländer hinweg einheitlich sind und/oder nicht einheitlich kommuniziert werden und/oder sich schnell ändern, woraus Konfusion, Unsicherheit und/oder Ärger resultiert
Atmosphäre in Schulen	Ungewohnte/ungewollte Schulatmosphäre bei Schulöffnung	„Ich war wieder in der Schule. Irgendwie war die Atmosphäre ganz merkwürdig … so sollte Schule nicht sein …“	In einem Tweet wird die (ungewohnte) Atmosphäre in den Schulen nach den Wiedereröffnungen der Schulen unter Infektionsschutzmaßnahmen thematisiert
